# Social divisions and risk perception drive divergent epidemics and large later waves

**DOI:** 10.1017/ehs.2023.2

**Published:** 2023-02-23

**Authors:** Mallory J. Harris, Kimberly J. Cardenas, Erin A. Mordecai

**Affiliations:** Biology Department, Stanford University, Stanford, CA 94301, USA

**Keywords:** infectious disease, social division, compartmental model, awareness, risk perception

## Abstract

During infectious disease outbreaks, individuals may adopt protective measures like vaccination and physical distancing in response to awareness of disease burden. Prior work showed how feedbacks between epidemic intensity and awareness-based behaviour shape disease dynamics. These models often overlook social divisions, where population subgroups may be disproportionately impacted by a disease and more responsive to the effects of disease within their group. We develop a compartmental model of disease transmission and awareness-based protective behaviour in a population split into two groups to explore the impacts of awareness separation (relatively greater in- vs. out-group awareness of epidemic severity) and mixing separation (relatively greater in- vs. out-group contact rates). Using simulations, we show that groups that are more separated in awareness have smaller differences in mortality. Fatigue (i.e. abandonment of protective measures over time) can drive additional infection waves that can even exceed the size of the initial wave, particularly if uniform awareness drives early protection in one group, leaving that group largely susceptible to future infection. Counterintuitively, vaccine or infection-acquired immunity that is more protective against transmission and mortality may indirectly lead to more infections by reducing perceived risk of infection and therefore vaccine uptake. Awareness-based protective behaviour, including awareness separation, can fundamentally alter disease dynamics.

**Social media summary:** Depending on group division, behaviour based on perceived risk can change epidemic dynamics & produce large later waves.

## Graphical abstract

**Figure:**
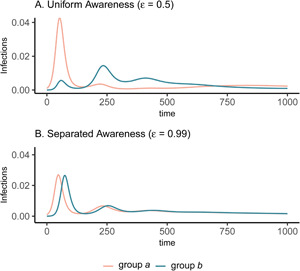

## Introduction

When an infectious disease causes substantial disease burden and death, people may perceive their risk of infection based on their awareness of the magnitude of disease-linked outcomes and respond by modifying their behaviour (An et al., 2020; Cheok et al., 2021; Gidengil et al., 2012; Ridenhour et al., 2022; Yan et al., 2021). In turn, protective behaviours like physical distancing, mask wearing and vaccination may suppress transmission, reducing peak and total infections and disease-linked mortality (Abaluck et al., 2022; Toor et al., 2021; Yan et al., 2021). Awareness-based behaviour describes protective measures that are adopted in response to epidemic intensity. Bidirectional feedback between protective behaviour and epidemic intensity can lead to unexpected and nonlinear dynamics, such as plateaus and oscillations in cases over time, if protective measures are abandoned over time (e.g. fatigue with non-pharmaceutical interventions may lead to a regular decline in adherence) or the strength of protection wanes (e.g. waning immunity from vaccination or infection) (Arthur et al., 2021; Eksin et al., 2017; Perra et al., 2011; Weitz et al., 2020). Models that split the population into categories with respect to the disease (i.e. compartments) and mathematically define transition rates between different states are widely used to understand such complex epidemic dynamics. Compartmental models may incorporate awareness as a function of deaths or cases that reduces transmission evenly across the population (Arthur et al., 2021; Weitz et al., 2020). However, real populations are sharply divided in physical interactions, demography, ideology, education, housing and employment structures, and information access. These social divisions can impact the transmission of both pathogens and information within and between groups, altering epidemic dynamics. The impacts of such asymmetrically spreading disease and awareness in a highly divided population are not well understood (Acevedo-Garcia, 2000; Farmer, 1996; Grief & Miller, 2017).

Populations may be subdivided based on an array of factors (e.g. race, ethnicity, age and geography), with marked differences in pathogen exposure and infection severity (Farmer, 1996; Greene et al., 2015; Li et al., 2016; Poteat et al., 2020; Williams & Cooper, 2020; Zelner et al., 2020). Risk of pathogen introduction may vary between groups: high-income groups may encounter pathogens endemic to other regions through international travel, low-income groups may have heightened likelihood of exposure connected to poor housing quality and insufficient occupational protections, and certain regions and occupations experience greater risks of exposure to zoonotic illnesses (Benfer et al., 2021; Cubrich, 2020; Dhewantara et al., 2018; Greene et al., 2015; Pramasivan et al., 2021). Once a pathogen is introduced, it may spread at different rates within groups based on factors like housing density and access to healthcare (Benfer et al., 2021; Poteat et al., 2020; Quinn et al., 2011). Further, the severity of infection may vary directly with group identity owing to underlying biological differences (e.g. age or sex), as a function of co-morbidities especially prevalent in one group owing to underlying inequities (e.g. lung disease connected to environmental pollution or heart disease associated with factors driven by structural racism), or through heterogeneity in access to and quality of healthcare (Calvin et al., 2003; Lane et al., 2022; Li et al., 2016; Poteat et al., 2020; Quinn et al., 2011; Takahashi et al., 2020; Williams & Cooper, 2020; Wu et al., 2020). Mixing, or between-group contact rates, can alter transmission dynamics. Physical barriers (e.g. geographic boundaries, schools, residential segregation and incarceration) and preferential contact with members of one's own group may reduce interactions and subsequent transmission between groups, a characteristic we describe as *separated mixing* (Arnold et al., 2022; Doherty et al., 2009; Greene et al., 2015; Harris et al., 2021; Rothenberg et al., 2005). Infectious disease models that account for differences in vulnerability within subgroups of a population and separated mixing can help to illustrate the emergence of health inequities and justify structural interventions to reduce these disparities (Jacquez et al., 1988; K. C. Ma et al., 2021; Richardson et al., 2021; Zelner et al., 2022). However, such models may miss an important behavioural dimension by failing to account for variation in awareness-based behaviour changes among groups.

Awareness and behavioural heterogeneity can significantly alter disease dynamics: for example, protective behaviour adoption based on disease status of social connections may slow pathogen transmission, while social clustering in vaccine exemptions may lead to outbreaks (Funk et al., 2009; Herrera-Diestra & Meyers, 2019; Omer et al., 2008). Personal perception of disease severity may be influenced by population-level social norms and mass media, regardless of group identity. However, attitudes toward diseases and protective behaviours may also vary considerably between groups and correspond to actual risk and personal experiences of close social ties with the disease (Anthonj et al., 2019; Brug et al., 2004; Christensen et al., 2020; Holtz et al., 2020; Oraby et al., 2014; Simione & Gnagnarella, 2020). While prior awareness-based models have examined outcomes given different scales of information (i.e. local or global), we aim to characterize risk perception based on group-level information in a population split into two distinct and well-defined groups (Funk et al., 2010). We define *separated awareness* as greater in- vs. out-group awareness of current epidemic conditions in a split population. We predict that, by producing behavioural responses more reflective of each group's risk, separated awareness may reduce differences between groups in disease burden that might otherwise occur (Steinegger et al., 2022). Understanding the impacts of separation with respect to mixing and awareness on disease dynamics may be important for characterizing differences in epidemic burden and effectively intervening to mitigate population inequities (K. C. Ma et al., 2021; Richardson et al., 2021; Steinegger et al., 2022; Weston et al., 2018; Zelner et al., 2022).

Here, we investigate the impacts of intergroup divisions on epidemic dynamics using an awareness-based model for transmission of an infectious disease, in which adoption of protective measures (either non-pharmaceutical interventions or vaccinations) is linked to recent epidemic conditions and mediated by awareness.

We ask:

1. How do separated awareness and mixing interact to affect differences between groups in epidemic dynamics?
2. How does fatigue interact with awareness separation to affect long-term epidemic dynamics?
3. When vaccines are introduced, how does immunity interact with awareness separation to affect long-term epidemic dynamics?

## Methods

### Non-pharmaceutical intervention model

We model disease transmission with awareness-based adoption of non-pharmaceutical interventions that reduce transmission rates. See Supplementary Figure S1 for a compartmental diagram for this model and Supplementary Table S1 for parameter definitions. We model disease transmission with a Susceptible–Infectious–Recovered–Deceased model, tracking the proportion of the population in each compartment through time. Susceptible individuals have never been infected or vaccinated. New infections arise through contact between susceptible and infected individuals, with the transmission coefficient *β* describing the rate at which the pathogen spreads. Individuals exit the infected compartment at per capita rate *ρ*, the inverse of infectious period 1/*ρ* and either recover or die. The fatality probability, or fraction of individuals exiting the infectious compartment who die, is *μ* (meaning that recovery after infection occurs with probability 1 − *μ*). In this model, recovered individuals have durable immunity and cannot be reinfected. The initial model does not include vaccine-derived immunity, an extension we consider below (Equation 3).

We further categorize the population based on whether they adopt behaviour that is Protective (P) or Unprotective (U). Compartment names contain two letters, the first indicating disease status and the second indicating behaviour (e.g. SU denotes Susceptible people with Unprotective behaviours). We track the behavioural status of Recovered and Deceased individuals (at the time of death), although they do not contribute directly to transmission. Protective measure efficacy against infection is determined by a scaling factor *κ* describing the degree to which the behaviour prevents infection (where *κ* = 0 corresponds to complete protection and *κ* = 1 corresponds to no protection). Protective measures affect the behaviour of both susceptible and infected individuals, so transmission rate is reduced by a factor of *κ*^2^ in encounters where both parties have adopted protective measures. Living individuals can switch between protective and unprotective behaviour, and we assume that the rates of these behavioural transitions are independent of their own disease status. Unprotective individuals adopt protective behaviours based on awareness (*α*(*t*), Equation 2), or perceived epidemic intensity at a given point in time. Awareness is the product of disease-induced deaths over the past ℓ days (making ℓ a measure of memory) and a responsiveness constant *θ*. Protective behaviours are abandoned owing to fatigue at per capita rate *ϕ*.

To study the impact of social divisions, we further split the population into two groups of equal size, where group membership is fixed, and each group contains all epidemiological and behavioural compartments. The groups are labelled as *a* and *b* and indicated as a subscript in compartment names (e.g. *SU*_*a*_ corresponds to the prevalence of Susceptible–Unprotective individuals in group *a*). We arbitrarily designate group *a* as having greater underlying vulnerability to infection or disease-linked mortality in all of the following scenarios. Parameters may vary between groups, as indicated by subscripts (e.g. *θ*_*a*_ corresponds to responsiveness in group *a*). If parameters are equivalent for both groups, we exclude the subscript (e.g. *θ* = *θ*_*a*_ = *θ*_*b*_).

Preferential within-group mixing is represented by homophily parameter *h*, corresponding to the proportion of contacts that are within-group. When *h* is 0.5, mixing is *uniform*, meaning that individuals are equally likely to contact members of their own group as members of the opposite group. As *h* approaches 1, mixing becomes increasingly separated, meaning that contacts are increasingly concentrated within groups. Similarly, we consider separation in awareness, 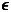, or the relative weight of in-group vs. out-group awareness of deaths for protective behaviour.

The system of equations for group *a* is as follows (equations for group *b* can be derived symmetrically):1
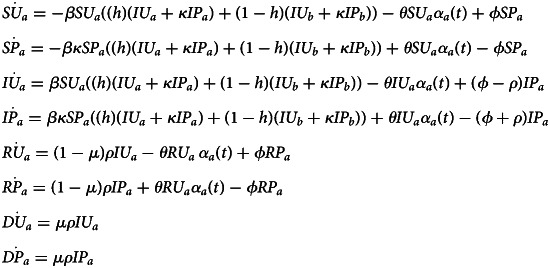
where *α*_*a*_(*t*) is the awareness equation for group *a*:2
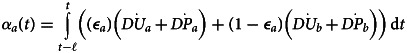


### Vaccination model

We develop an alternative model in which the awareness-based behaviour is vaccine uptake, rather than non-pharmaceutical interventions. See Supplementary Figure S2 for a compartmental diagram for this model and Supplementary Table S1 for parameter definitions. Here, the second letter of compartment names indicates immune status: Unprotective (U), Transmission and Mortality-reducing Immunity (T), or Mortality-reducing Immunity (M). This reflects our assumption that immunity initially reduces both transmission and mortality following infection or vaccination, and later wanes to reduce mortality but not infection.

As in the non-pharmaceutical intervention model, susceptible people without prior immunity (SU) may become infected and then recover or die according to baseline infection parameter values. Susceptible individuals may become vaccinated and transition directly to the recovered compartment, bypassing infection, at a rate dependent on awareness. There may be a lag between the beginning of the epidemic and vaccine introduction at time point *t*_*v*_ (Supplementary Figures S13 and S14). To evaluate long-term immune effects of vaccination and infection on epidemic dynamics, we incorporate waning immunity by including distinct T and M compartments, as described above.

After vaccination or infection, individuals temporarily have complete protection from infection (RT). At per capita rate *ω*, they regain susceptibility to infection, this time with transmission and mortality-reducing immunity (i.e. *ST*). As in the non-pharmaceutical intervention model, transmission-reducing protection scales transmission rates for susceptible and infected individuals by a constant. Additionally, immunity reduces disease-linked mortality by scaling factor *ζ*. Transmission-reducing immunity is lost at per capita rate *ϕ*, while mortality-reducing immunity is retained over the course of the simulation, reflecting how neutralizing antibody production may decay over time while cellular immune responses are more durable (Siggins et al., 2021). Susceptible individuals with mortality-reducing immunity alone (*SM*) may regain transmission-reducing immunity via vaccination, which occurs based on the same awareness function as vaccination of people without immune protection.

The system of equations for this model in a population without groups is:3
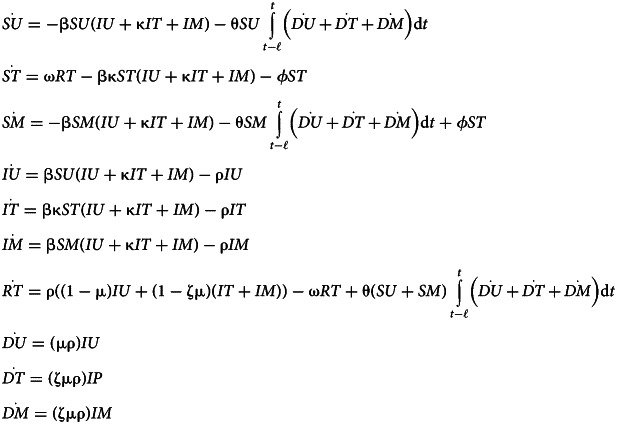


The equations for a split population with separated mixing and awareness can be derived following Equation (1).

### Simulations

We ran simulations in R version 4.0.2, using the dede function in the deSolve package, which solves systems of differential equations (Soetaert et al., 2010). The population begins as almost fully susceptible (*S*(0) ≈ 1), with a small initial infection prevalence (*I*(0)) to seed the outbreak and no protective behaviours. In the non-pharmaceutical intervention scenarios (scenario 1 and 2), the sole initial difference between groups is caused by introducing the pathogen into group *a* alone at prevalence *I*_*a*_(0) = 0.001. In the vaccination scenario (scenario 3), the pathogen is introduced in both groups at prevalence *I*(0) = 0.0005 and the fatality probability for group *a* is twice that of group *b* (*μ*_*a*_ = 0.02 and *μ*_*b*_ = 0.01). An interactive R Shiny app that allows users to simulate epidemics for the non-pharmaceutical intervention model across parameter values is available at https://mallory-harris.shinyapps.io/divided-disease/.

## Results

### 1. Separated mixing and awareness

To understand how separation in awareness and mixing interact to alter short-term epidemic dynamics in a split population, we model awareness-based adoption of non-pharmaceutical interventions (Equation 1); all model parameters are defined in Supplementary Table S1 and a compartmental diagram is provided as Supplementary Figure S1. As described above, the pathogen is introduced in group *a* alone; all other parameters are equivalent between groups. To simplify short-term awareness-based behaviour, this scenario does not incorporate memory or fatigue (ℓ = 1 and *ϕ* = 0). First, we allow both mixing (*h*, which drives the contact and contagion process) and awareness (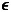, which drives protective behaviour adoption) to be either uniform (functioning like a single population) (0.5) or highly separated (0.99).

**Figure 1.**
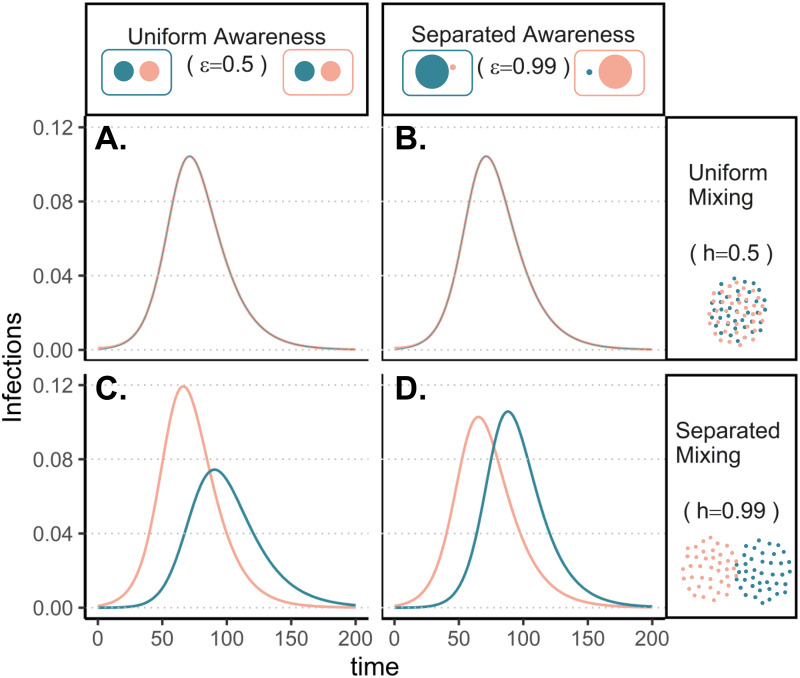
Epidemic peaks are offset in time between groups when mixing is separated (C, D), and in magnitude when awareness is uniform but mixing is separated (C). Plots show the prevalence of infections over time in group *a* (pink) and group *b* (green) under four scenarios: awareness is uniform (A, C; 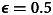) or separated (B, D; 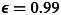); mixing is uniform (A, B; *h* = 0.5) or separated (C, D; *h* = 0.99). We assume the pathogen is introduced only in group *a* at prevalence 0.001 and that all other parameters are equivalent between groups: transmission coefficient (*β* = 0.2), infectious period (1/*ρ* = 10), fatality probability (*μ* = 0.01), protective measure efficacy (*κ* = 0.3), responsiveness (*θ* = 100), memory (ℓ = 1), and fatigue (*ϕ* = 0). Lines overlap under uniform mixing (top row).

The groups experience identical epidemic dynamics regardless of awareness separation when mixing is uniform (Figure 1A, B), as the pathogen introduced into group *a* quickly spreads into group *b* and circulates evenly within and between groups. When groups mix separately, differences in epidemic dynamics between groups arise and depend on awareness separation (Figure 1C, D). Therefore, we focus the rest of our analyses on cases where mixing is separated to examine the impacts of awareness separation. When awareness is uniform, epidemic shape differs in both timing and magnitude between groups, increasing the peak size and total infections in the more vulnerable (earlier epidemic introduction) group *a* and decreasing both in group *b* (Figure 1C). Group *a* also has more cumulative deaths than group *b* under uniform awareness, while cumulative deaths across the full population (group *a* and group *b* combined) are approximately constant across different levels of awareness and mixing separation (Supplementary Figure S3).

**Figure 2.**
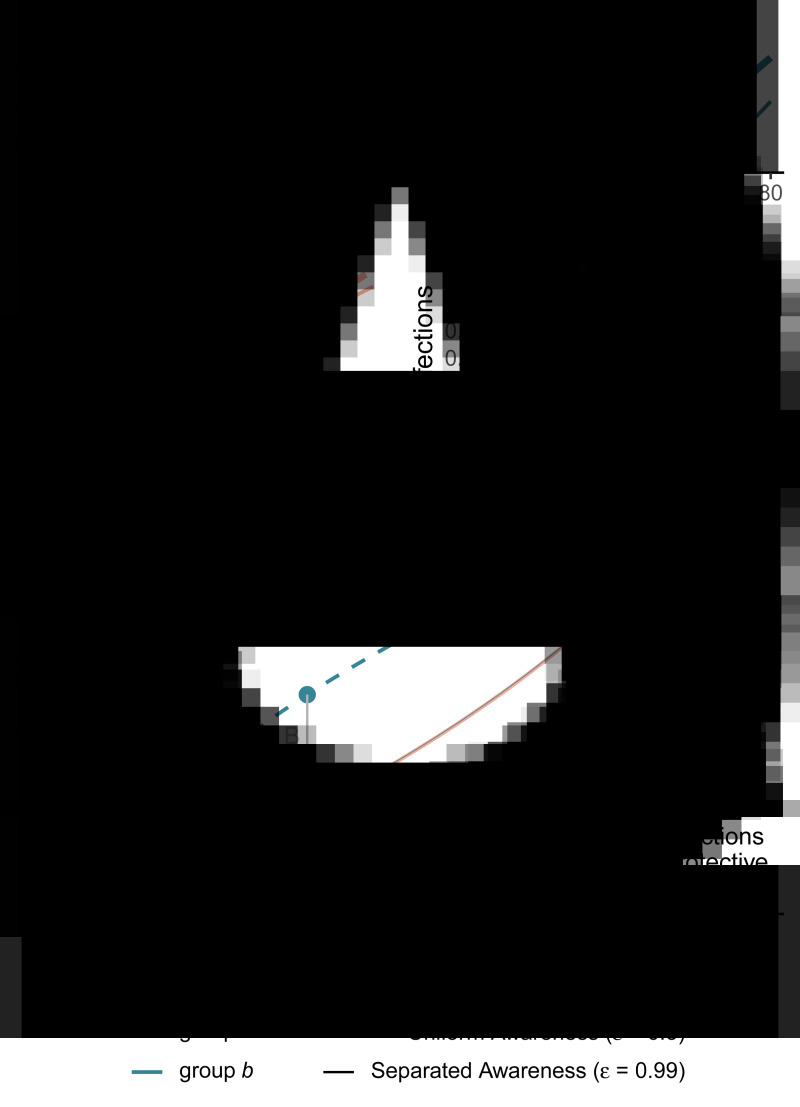
Separated awareness reduces between-group differences by reducing group *b*'s awareness of the emerging epidemic and augmenting group a's response to the introduction of the pathogen. We initialize our model using the same parameters as Figure 1 with separated mixing (*h* = 0.99). We compare uniform awareness (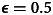; dashed lines) and separated awareness (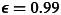; solid lines). At the top, we compare early time series (through *t* = 80) of (A) protective attitude prevalence in group *a*; (B) protective attitude prevalence in group *b*; (C) cumulative infections in group *a*; (D) cumulative infections in group *b*. (E) A phase portrait of protective attitude prevalence against cumulative infections in group a (pink) and group *b* (green). Points indicate values at *t* = 80, corresponding to the end of the time series in (A–D). Arrows indicate differences in protective attitude prevalence (grey) and cumulative infections (black) at *t* = 80 for separated vs. uniform awareness, with letters corresponding to time series panel labels.

Awareness separation changes epidemic size in both groups by modulating how quickly protective behaviour arises relative to pathogen spread (Figure 2). Uniform awareness reduces total infections in group *b*, which adopts protective behaviour by observing mortality in group *a* at a point when infections within group *b* remain relatively low (Figures 1C and 2B, D, E). Meanwhile, uniform awareness causes group *a* to underestimate disease severity owing to the lack of early mortality in group *b*, leading to decreased early protective behaviour and a larger outbreak (Figures 1C and 2A, C, E). When awareness is separated, group *b* has little awareness of the emerging epidemic localized to group *a*, while group *a* responds to its relatively higher early disease burden with increased awareness, driving epidemic dynamics between the two groups to be similar in shape but delayed in time for group *b* (Figure 1D). Therefore, awareness separation reduces the differences between groups in epidemic shape (e.g. peak size, total infections), while mixing separation offsets them in time (Figure 1C, D, Supplementary Figures S4 and S5).

Differences between groups in epidemic dynamics only arise at high levels of mixing separation (*h* > 0.9) but can occur at intermediate levels of awareness separation (Supplementary Figures S4 and S5; e.g. 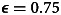). Awareness separation also reduces differences between groups in severe outcomes when groups differ in their transmission coefficients, infectious periods, or fatality probabilities (Supplementary Figures S6–8).

### 2. Fatigue and awareness separation

We introduce memory and fatigue to examine the long-term impacts of separated awareness when awareness-driven protective behaviour is abandoned over time. Once again, the pathogen is introduced into group *a* alone and all other parameters are equivalent between groups. To maintain between-group differences, we assume separated mixing (*h* = 0.99).

**Figure 3.**
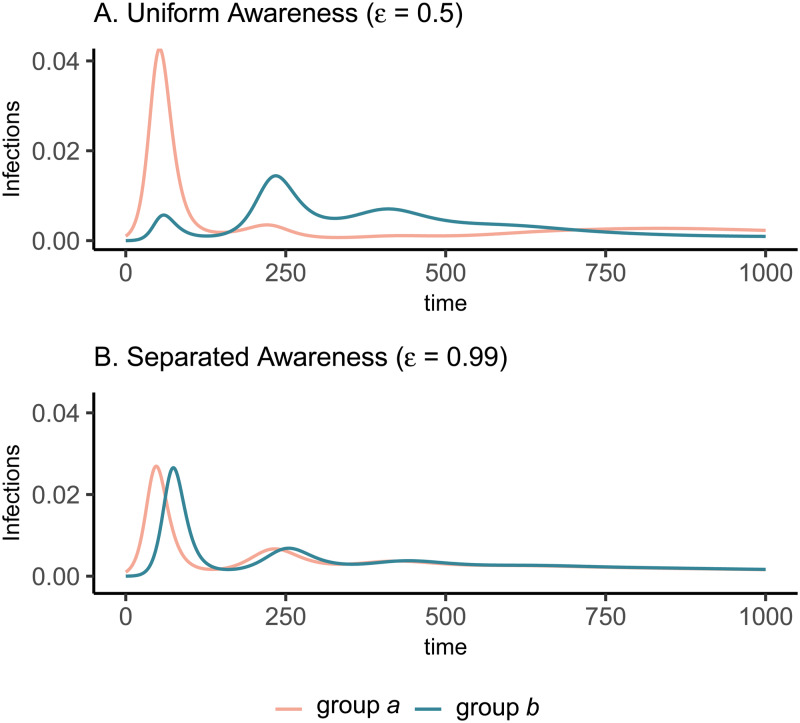
Fatigue and long-term memory produce multiple epidemic peaks, which exceed the size of the initial peak in group b when uniform awareness and separated mixing leave that group with a high proportion of susceptible people following the first wave. We initialize the model with separated mixing (*h* = 0.99), long-term memory (ℓ = 30), and fatigue (*ϕ* = 0.02); all other parameters are the same as in Figure 1. We consider infections in group a (pink) and group b (green) over a longer time period (1000 days, compared with 200 days in Figure 1). The panels correspond to (A) uniform awareness (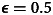) and (B) separated awareness (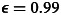).

In all cases, when protective behaviour wanes with fatigue, three distinct peaks emerge before transmission plateaus at low levels and declines gradually (Figure 3). The initial difference between groups with uniform awareness means that group *b* retains a relatively larger proportion of susceptible individuals who avoided infection in the first wave by rapidly adopting protective behaviours (Figures 1C and 3A). As a result, the second and third waves in group *b* exceed the first wave in peak and total infections (Figure 3A). Meanwhile, uniform awareness causes the second and third waves in group *a* to be smaller compared with separated awareness (Figure 3A vs. B). Under uniform awareness, the third wave in group *a* is considerably delayed, peaking around 800 days (vs. 450 days under separated awareness). At intermediate awareness separation (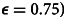, the first and second waves in group *b* are approximately equivalent in size (Supplementary Figure S10). As shown in the case without memory and fatigue (Figure 1), when both mixing and awareness are separated, the groups differ mainly in the timing of epidemic peaks rather than in their magnitude, before converging on a long and slow decline (i.e. shoulder; Figure 3B). In the full population, awareness separation may change infection prevalence over time but has no impact on cumulative deaths (Supplementary Figure S11).

### 3. Immunity and awareness separation

Next, we consider the implications of awareness-based vaccine uptake in a split population given waning immune protection against infection and durable protection against mortality (Equation 3, Supplementary Figure S2). We model immunity from prior infection as equivalent to immunity from vaccination. Unlike in the previous analyses, the pathogen is now introduced at the same prevalence in both populations simultaneously to ensure that groups *a* and *b* begin the post-vaccine period with similar levels of immunity. Group differences are driven by a fatality probability in group *a* that is twice that of group *b*. Again, we assume separated mixing (*h* = 0.99) to maintain distinct dynamics between the groups. We initiate vaccination at 200 days, after an initial large wave of infections. Our analyses focus on the period following the introduction of vaccines to understand how awareness separation modulates the impact of this protective measure across a period where infection is already well established in both populations but substantial proportions of the population remain susceptible.

**Figure 4.**
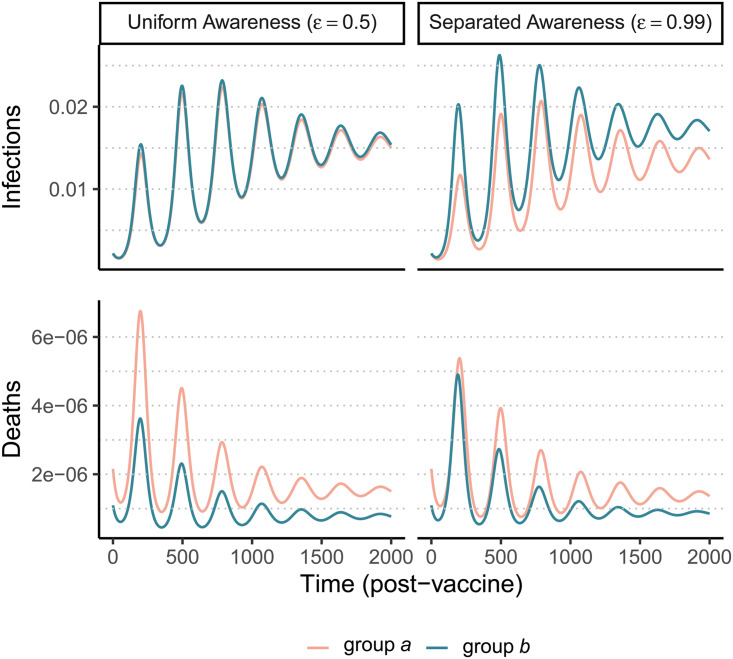
Waning immunity and awareness-based vaccination drive epidemic cycles; separated awareness reduces the disparity in deaths (C vs. D) as more vulnerable group *a* members become vaccinated at a higher rate. We consider infections (A, B) and deaths (C, D) in the post-vaccine period in group *a* (pink) and group *b* (green) where the fatality probability for group *a* is double that of group b (*μ*_*a*_ = 0.02 and *μ*_b_ = 0.01). The x-axis gives time since vaccination began (t_v_ = 200). We compare uniform awareness (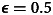) (A, C) and separated awareness (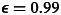) (B, D). Other parameter values are: *β* = 0.2 (transmission coefficient), *κ* = 0.05 (transmission-reducing immunity), *ζ* = 0.05 (mortality-reducing immunity), *ω* = *ϕ* = 0.01 (waning immunity), infectious period (1/ρ = 10), *θ* = 20 (responsiveness), ℓ = 30 (memory), *h* = 0.99 (separated mixing), *I*_0_ = 0.0005 (initial infection prevalence). See Supplementary Figure S12 for a time series plot including the pre-vaccine period.

After an initial large wave (displayed in Supplementary Figure S12), vaccination and waning immunity lead to damped cycles of infections and deaths (Figure 4). As was the case with the non-pharmaceutical intervention model (Figure 1), when awareness drives vaccination behaviour, separated awareness helps to reduce differences in mortality between groups (Figure 4D vs. C). Group *a* becomes vaccinated at a higher rate in response to the greater number of deaths observed in group *a*, an effect that is most notable during the second epidemic peak following vaccine introduction (Figure 4D). Therefore, group *a* also has fewer infections than group *b* in later waves under separated awareness (Figure 4B), while the two groups experience identical infection dynamics (despite the larger disparity in deaths) given uniform awareness (Figure 4A).

**Figure 5.**
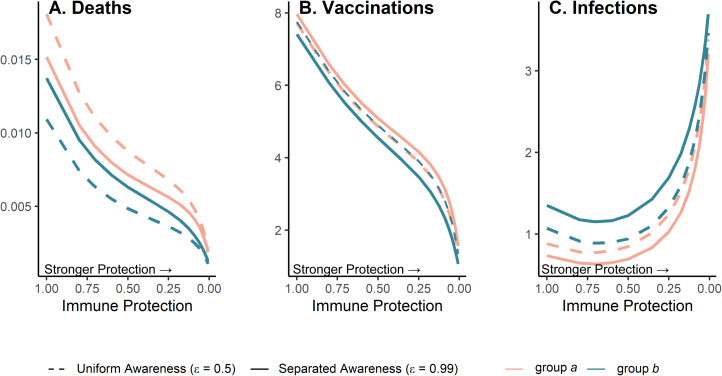
Greater immune protection (from vaccination and infection) leads to lower death rates (A), which in turn decreases vaccination rates (B) and increases infection rates (C); separated awareness reduces disparities in death rates (A) as groups are vaccinated at different rates proportional to their risks of death (B), creating differences in infection rates (C). We vary immune protection, defined as transmission-reducing immunity and mortality-reducing immunity, where both parameters are assigned the same values (*κ* = *ζ*). We assume immune protection is equivalent for vaccine- and infection-derived immunity. The x-axis is reversed because smaller values indicate stronger protection. We examine the impacts of stronger immune protection (lower values of *κ* and *ζ*) on total deaths (A), vaccinations (B) and infections (C) in the post-vaccine period (t = 200 through t = 2200). We consider the post-vaccine period to focus on the impacts of an awareness-based intervention administered under different levels of awareness separation. We compute each quantity for group a (pink) and group b (green) given uniform (dashed lines; 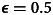) or separated (solid lines; 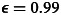) awareness. Other parameter values are the same as Figure 4.

Because vaccination protects against infections and deaths, and recent deaths feed back to influence awareness-driven vaccine uptake, there is a potential tradeoff between immune protection from vaccines and epidemic dynamics. We explored this tradeoff by examining the effect of variation in immune protection on epidemic dynamics and their feedbacks on vaccine uptake rate, assuming that immune protection causes the same proportional reduction in transmission and mortality (*κ* = *ζ*). As expected, greater immune protection reduces the number of deaths by directly reducing the fatality probability. However, because of awareness-driven vaccine uptake, vaccination can produce diminishing returns at the population scale where doubling immune protection from death and infection only reduces total deaths by about one eighth owing to the compensatory reduction in vaccine uptake (Figure 5A), despite doubling individual protection for vaccinated people. Since a more effective immune response reduces mortality, the perceived risk associated with infection declines and fewer people become vaccinated (Figure 5B). The tradeoff between the direct impacts of immune protection on preventing infections and reduced uptake produces a nonlinear relationship between total infections and immune protection (Figure 5C). At low immune protection, infections remain approximately constant as immune protection improves. At higher levels of immune protection, reduced uptake with stronger protection leads to more infections (Figure 5C).

Separated awareness drives differences between groups in vaccination behaviour – the higher-risk group *a* gets vaccinated at a higher rate in response to awareness of the greater cumulative mortality in that group (Figure 5B). This in turn increases differences in infections (group *a* experiences lower infection rates; Figure 5C) but decreases differences in mortality between groups (death rates are lower for group *a* but higher for group *b* than in the uniform awareness scenario; Figure 5A). Since group *a* is at a higher inherent risk of mortality given infection, separated awareness differentially promotes vaccination and reduces infection in this group, while uniform awareness causes group *a* to ignore its higher risk of mortality (Figure 5A, B, solid vs. dashed lines). Cumulative deaths increase especially quickly during the initial wave absent vaccination because the population lacks transmission- or mortality-reducing immunity. When vaccination begins earlier in the epidemic (prior to the initial peak around *t* = 100), separated awareness has greater potential to reduce the difference in cumulative deaths between the two groups (Supplementary Figures S13 and S14). Early vaccination may also reduce cumulative deaths and infections in each group (Supplementary Figure 14).

## Discussion

Awareness separation and social divisions may interact to fundamentally alter disease dynamics, creating or erasing differences between groups in the timing and magnitude of epidemic peaks. Uniform awareness can exacerbate differences between population subgroups when the more vulnerable group (e.g. the group where the pathogen is introduced or the group with higher fatality probabilities) underestimates the in-group risk of disease and fails to adopt early protective measures (Figures 1 and 5). At the same time, the initially less-vulnerable group receives indirect protection from observing and responding to epidemic effects in the more vulnerable group, adopting protective measures that reduce their total and peak infections (Figures 1 and 5). However, when awareness-driven behaviour fades with fatigue, the relative disease burden may shift between groups such that the group that initially had fewer infections has relatively more infections in subsequent waves, especially when uniform awareness protects the initially less-vulnerable group during the first wave of infection (Figure 2). Awareness separation diminishes between-group differences in severe outcomes (Figures 1–5, Supplementary Figures S6–S8), but may do so by increasing differences in behaviour and infections (Figures 4 and 5, Supplementary Figure S8). For example, when the more vulnerable group has a higher rate of disease-linked mortality, awareness separation leads them to have higher vaccine uptake in response to their heightened perceived (and actual) risk, narrowing the difference in mortality (Figure 5). More broadly, awareness separation generally reduces differences in severe outcomes between groups by producing preferential uptake of preventative measures by the group with the greatest recent mortality, which is usually the group at greatest current risk.

In this model, greater awareness separation generally reduces differences in severe outcomes between groups, but the magnitude of these impacts may vary depending on disease properties (e.g. transmission coefficient) and behavioural and social processes (e.g. responsiveness to disease-linked mortality) (Supplementary Figures S9, S13 and S14). Outcomes may be further modulated by public health orders and the timing of different interventions. For example, there is greater potential for awareness separation to reduce between-group differences in mortality given earlier vaccine introduction (Supplementary Figures S13 and S14). The existing models could be modified to incorporate population-wide measures, particularly time-limited non-pharmaceutical intervention mandates, to study how social and behavioural processes may shift the optimal timing of interventions in the full population or either group (Ketcheson, 2021; Morris et al., 2021). Although this model and others assume that protective behaviour uptake is independent of disease status (Mehta & Rosenberg, 2020; Smaldino & Jones, 2021), the model could be modified to link behaviour with known disease status (e.g. accelerated uptake of or reduced fatigue with protective measures by people with symptomatic infections) (Eksin et al., 2017; Funk et al., 2009). To assess the robustness of our conclusions about the effects of awareness separation, the same scenarios could be evaluated across different models of awareness-based behaviour changes, including saturation at a certain threshold for deaths (Weitz et al., 2020), consideration of both lethal and non-lethal impacts of disease (e.g. hospitalizations and cases), or optimization to balance the benefits of protection against the costs of various measures (Arthur et al., 2021; Barrett et al., 2011; Eksin et al., 2017). The latter approach may clarify a point that is not addressed in our analysis: although awareness separation may reduce disparities in severe disease-linked outcomes, this phenomenon is not necessarily equitable or desirable. In fact, if self-protection is associated with significant costs, already-vulnerable populations may suffer compounding costs as they balance self-protection against significant disease risk without adequate support from a broader community that does not share their risks (Atchison et al., 2021; Barrett et al., 2011; Jay et al., 2020; Skinner-Dorkenoo et al., 2022). Further, structural inequities often leave population subgroups that are vulnerable to larger, more severe outbreaks with reduced access to protective measures like health education, treatment, vaccination, and paid leave (Cardona et al., 2021; Christensen et al., 2020; Clouston et al., 2015; Dryhurst et al., 2020; Heymann et al., 2021; Poteat et al., 2020; Ridenhour et al., 2022; Simione & Gnagnarella, 2020; Williams & Cooper, 2020). Resulting differences in rates of protective behaviour uptake and effectiveness can compound disparities between groups and reduce the protective impact of awareness separation for more-vulnerable groups.

Epidemics are complex phenomena that typically involve heterogeneous mixing among groups of people that differ in biological and social risk factors, dynamic evolution of host behaviour, pathogen infectiousness and immune evasion, and ever-changing epidemiological and policy responses to real and perceived risk. Despite this range of potential drivers, we show here that a simple model that captures two key social processes – awareness-driven protective behaviour in a split population that can be separated in mixing and awareness – can drive many of the complex dynamics observed in emerging epidemics like Covid-19. For example, when awareness is uniform and mixing is separated, the group in which the pathogen is introduced later can experience second and third waves that exceed the initial wave in size (Figure 3). This trend resembles one observed in the United States during the first year of the Covid-19 pandemic, where certain regions where the virus was introduced early (e.g. New York City metropolitan area) experienced a large early wave and relatively few infections over the rest of the year, while other regions (e.g. the southern United States) generally had small early waves and larger second and third waves. Many hypotheses have been introduced to explain this phenomenon (e.g. policy, seasonal climate factors, and population density) and several factors may have contributed to this pattern (Y. Ma et al., 2021; Sy et al., 2021). Yet in our model these dramatic differences among populations in epidemic waves occur despite the groups being identical in transmission rates and disease outcomes and are entirely due to awareness-driven behaviour with uniform awareness among groups (Figure 3). Although the current analysis does not examine causation, and observed trends during Covid-19 probably involved a confluence of drivers, we have demonstrated how a simple behavioural process can qualitatively reproduce complex epidemic dynamics observed in real populations. To understand the extent of awareness separation in real populations and the role of specific behavioural processes in observed trends, our model could be parameterized using a combination of epidemiological, survey, mobility and social media data (Chang et al., 2021; Shen et al., 2021; Weitz et al., 2020).

Feedback between vaccine efficacy and awareness-based vaccine uptake can also produce the counterintuitive scenario where vaccines that cause a greater reduction in transmission and mortality lead to more cumulative infections, even as deaths are reduced (Figure 5). If, as we assume here, protective behaviour is driven by awareness of severe outcomes like mortality, awareness separation may reduce differences in deaths between groups while widening differences in cases (Figures 4 and 5). The potential for awareness separation in vaccine uptake to reduce between-group differences in mortality is greatest when vaccination is introduced earlier in the epidemic, indicating that intervention timing may have health equity implications (Supplementary Figures S13 and S14). Accounting for awareness-based adoption of protective behaviour is therefore critical for understanding complicated epidemic dynamics such as plateaus and cycles (Figures 3 and 4), accurately deploying protective measures and assessing their impact across different diseases and population subgroups (Arthur et al., 2021; Steinegger et al., 2022; Weitz et al., 2020).

Here we have considered arbitrarily defined groups that can be separated in mixing and awareness but initially differ only in the timing of pathogen introduction (Figures 1–3), fatality probability (Figures 4 and 5, Supplementary Figure S8), pathogen transmission (Supplementary Figure S6) or infectious period (Supplementary Figure S7). Real social groupings may fall along a number of social, demographic and geographic lines, while the assumption of two distinct and identifiable groups may not fully capture relevant social dynamics. The most relevant groupings with respect to awareness and disease risk may also depend on the disease. For infectious diseases that are generally more prevalent and severe in children (e.g. pertussis and measles), risk may depend on age while awareness is split between parents of young children vs. adults without children or among parents with different sentiments towards childhood vaccination (Bhattacharyya & Bauch, 2010). In the context of Covid-19, disease burden and attitudes toward preventative measures (e.g. masks and vaccines) have differed markedly across age, socioeconomic status and race, and over time, demonstrating how intersecting and imperfectly overlapping identities may interact to determine attitudes, protective behaviours and risk (Maroko et al., 2020; Schulz et al., 2020; van Holm et al., 2020). Moreover, ideological and social factors that do not correspond directly to disease risk (e.g. political affiliation) may influence decision-making and cause the level of protective behaviour in certain subgroups to diverge sharply from their relative risk for severe disease, potentially overcoming the effects of awareness separation (Christensen et al., 2020; Grossman et al., 2020). This process could be incorporated into our model by splitting the population into additional groups with respect to a cultural contagion or (mis)information spread process and allowing protective measures to be adopted based on awareness or contact with protective in-group members and rejected through fatigue or aversion to protective measures displayed by the opposite group (Mehta & Rosenberg, 2020; Smaldino & Jones, 2021).

Although we assumed that awareness was directly proportional to recent mortality, external influences like partisanship (Christensen et al., 2020; Grossman et al., 2020), media coverage (Shanta & Biswas, 2020), misinformation (Lee et al., 2021) and policy (Yan et al., 2021) may alter the perception of risk or the adoption of protective measures at both the individual and group level. Group identification and assessment of relative risk may be unclear or inaccurate based on uncertainty at the beginning of the outbreak, misinformation about risk factors, a gradient in risk (e.g. increasing risk with age), lack of data stratification or unobserved risk factors. Attitudes based on one disease may carry over to another disease even if risk factors differ. Relative risk across groups may also vary across time and space, potentially leading to inaccurate assessment based on prior conditions: for example, a mild initial epidemic wave can mislead a group into believing they are inherently more protected and thereby relaxing protective behaviours. Cognitive interventions that increase the accuracy of individual risk perception, especially in high-risk groups, may help to reduce between-group differences in disease burden (Sinclair, Hakimi, et al., 2021; Sinclair, Stanley, et al., 2021).

Our model may also be extended to other scenarios involving a transmission process and collective behaviour, particularly social contagions like the spread of rumours and trends. Additional parameter space may be explored via the R Shiny interactive app accompanying this project, which currently only incorporates the non-pharmaceutical intervention model (https://mallory-harris.shinyapps.io/divided-disease/). Considering awareness separation as a social process that may interact with mixing, fatigue, waning immunity, pathogen evolution and pharmaceutical and non-pharmaceutical interventions may help to explain how humans are affected by and respond to infectious diseases in the presence of social divisions.

## Research transparency and reproducibility

Code used to conduct these analyses are available on Github at: https://github.com/mjharris95/divided-disease

An interactive R Shiny app based on the non-pharmaceutical intervention model is available at: https://mallory-harris.shinyapps.io/divided-disease/

## Supplementary material

For supplementary material accompanying this paper visit https://doi.org/10.1017/ehs.2023.2. click here to view supplementary material
