# Supplementary material for: Social divisions and risk perception drive divergent epidemics and large later waves
Source: Evol Hum Sci. 2023 Feb 23;5:e8. doi: 10.1017/ehs.2023.2 (PMC10426078; doi:10.1017/ehs.2023.2)
Supplement: Supplementary file 1 [file S2513843X23000026sup001.docx]

[Parameter dictionary 3](#_Toc121239027)

[Supplementary Table 1. Parameter dictionary providing parameter symbols, descriptions, and values for different scenarios 3](#_Toc121239028)

[Compartmental diagrams 4](#_Toc121239029)

[Supplementary Figure 1. Compartmental diagram for non-pharmaceutical intervention model that tracks status with respect to infection and attitude toward protective behaviors. 4](#_Toc121239030)

[Supplementary Figure 2. Compartmental diagram for vaccination model that tracks status with respect to infection and immune status. 5](#_Toc121239031)

[Cumulative deaths at different levels of awareness and mixing separation 6](#_Toc121239032)

[Supplementary Figure 3. Separated mixing causes differences in cumulative deaths between the two groups, which close over time under separated awareness. 7](#_Toc121239033)

[Intermediate levels of awareness and mixing separation 8](#_Toc121239034)

[Supplementary Figure 4. Infection time series at intermediate levels of mixing and awareness separation demonstrate that a high level of mixing separation is necessary to produce between-group differences in epidemic trajectories. 9](#_Toc121239035)

[Supplementary Figure 5. Given highly separated mixing, between-group differences in total and peak infection prevalence decrease 10](#_Toc121239036)

[Separated awareness helps to overcome different types of between-group differences 11](#_Toc121239037)

[Supplementary Figure 6. Separated awareness reduces differences in epidemic size between groups in epidemic size that arise from differences in transmission rates coupled with separated mixing. 12](#_Toc121239038)

[Supplementary Figure 7. Separated awareness reduces differences in epidemic size between groups in epidemic size that arise from differences in infectious period coupled with separated mixing. 13](#_Toc121239039)

[Supplementary Figure 8. Separated awareness reduces differences in mortality between groups arising from differences in their fatality probabilities and causes differences in infections between the groups 14](#_Toc121239040)

[Sensitivity to transmissibility, protective measure efficacy, and responsiveness 15](#_Toc121239041)

[Supplementary Figure 9. The ratio of total infections in group a versus group b increases with reduced pathogen transmissibility, greater protective behavior efficacy, or greater responsiveness. 17](#_Toc121239042)

[Long-term memory and fatigue 18](#_Toc121239043)

[Supplementary Figure 10. At intermediate levels of awareness separation, the first and second waves in group b have similar infection peak sizes. 18](#_Toc121239044)

[Supplementary Figure 11. In the full population, awareness separation alters the shapes of the first and second waves of infections but does not significantly change cumulative deaths over time. 19](#_Toc121239045)

[Time series with pre-vaccine period 20](#_Toc121239046)

[Supplementary Figure 12. Epidemic intensity is greatest in the pre-vaccine period compared to the post-vaccine period. 20](#_Toc121239047)

[Sensitivity of vaccination outcomes to timing of vaccine introduction 21](#_Toc121239048)

[Supplementary Figure 13. Between-group differences in cumulative deaths increase most quickly at the beginning of the epidemic and may be reduced with earlier vaccine introduction. 22](#_Toc121239049)

[Supplementary Figure 14. Earlier vaccine introduction decreases cumulative deaths in both groups, has minimal impact on cumulative vaccinations, and may decrease cumulative infections if immune protection is low to intermediate. 23](#_Toc121239050)

# Parameter dictionary

Supplementary Table 1. Parameter dictionary providing parameter symbols, descriptions, and values for different scenarios. The parenthetical numbers in the values column indicate the scenario where the parameter takes the given values (1: separated mixing and awareness; 2: fatigue and awareness separation; 3: immunity and awareness separation).

| Parameter | Description | Value |
| --- | --- | --- |
| $\beta$ | Transmission coefficient | 0.2 (1, 2, 3) |
| $1/\rho$ | Infectious period (days) | 10 (1, 2 ,3) |
| $\mu$ | Fatality probability | 0.01 (1,2);  $\mu_{a}=0.01$ and $\mu_{b}=0.02$(3) |
| $\kappa$ | Transmission and infection reduction with protective behavior | 0.3 (1, 2); 0.05 (3) |
| $\theta$ | Responsiveness | 100 (1, 2); 20 (3) |
| $\mathcal{l}$ | Memory | 0 (1); 30 (2, 3) |
| $\phi$ | Fatigue (NPI model)/waning transmission-reducing immunity (vaccine model) | 0 (1); 0.02 (2); 0.01 (3) |
| h | Homophily | 0.5 (uniform) or 0.99 (separated) (1);  0.99 (separated) (2, 3) |
| $\epsilon$ | Assortative awareness level | 0.5 (uniform) or 0.99 (separated) (1, 2, 3) |
| $\omega$ | Return to susceptibility (waning immunity; 1/days) | 0.01 (3) |
| $\zeta$ | Mortality reduction | 0.05 (3) |
| $t_{v}$ | Vaccination Start Time (days) | 200 (3) |

# Compartmental diagrams


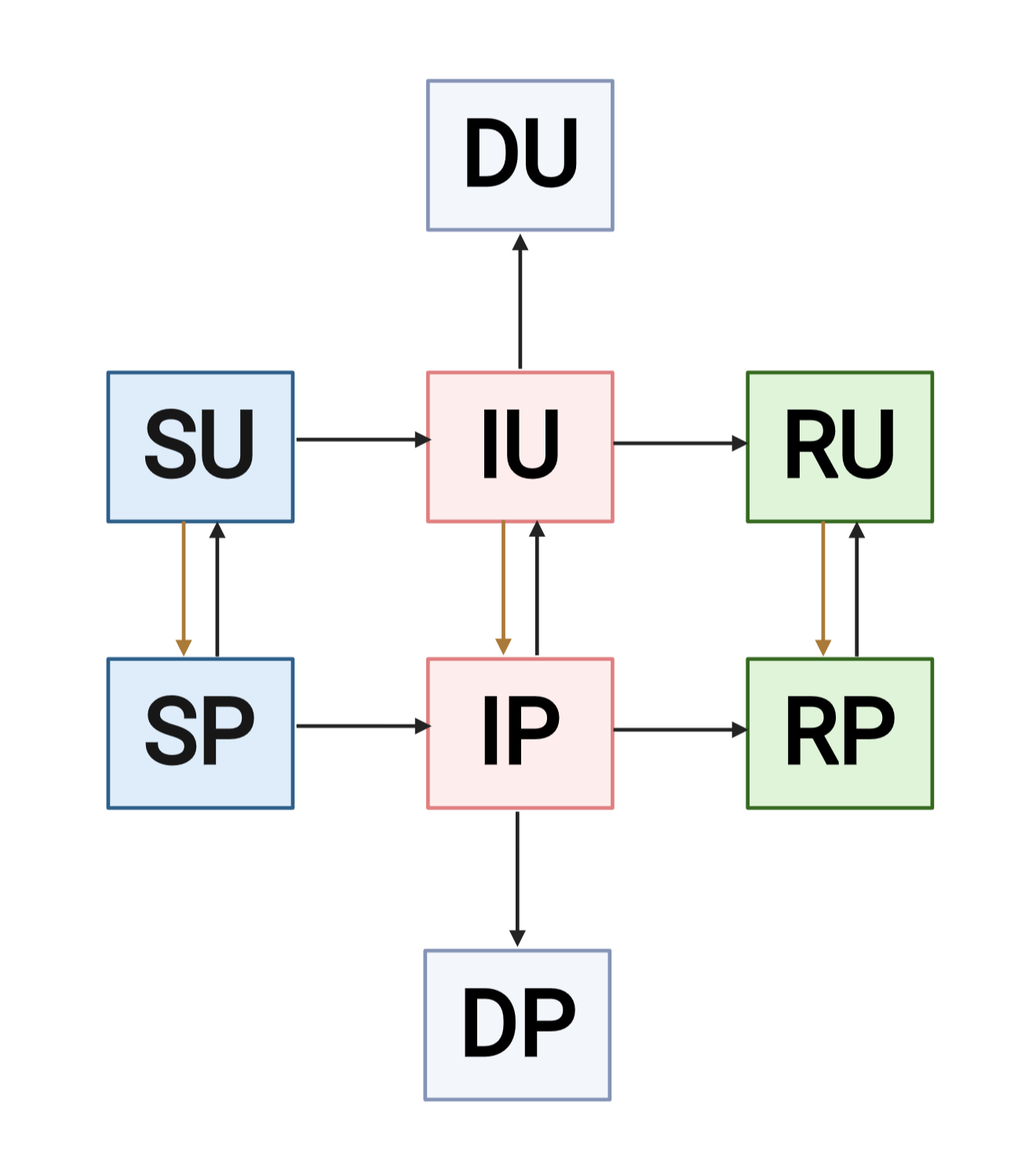


Supplementary Figure 1. Compartmental diagram for non-pharmaceutical intervention model that tracks status with respect to infection and attitude toward protective behaviors. The first letter of each compartment name gives the state with respect to the disease transmission process (S = Susceptible, I = Infectious, R = Recovered, D = Deceased) and the second letter of each compartment name gives state with respect to awareness-driven protective behavior (U = Unprotective, P = Protective). Squares are colored based on state with respect to disease. Potential transitions are indicated with arrows. Brown arrows indicate awareness-based adoption of protective measures while black arrows indicate other transitions, specifically disease state transitions and abandonment of protective measures with fatigue. This diagram corresponds to the model described in Equation 1 without groups. Each group contains the compartments and transitions displayed. Transitions between groups a and b are not possible, but transition rates in one group may be dependent on infection prevalence and mortality in the opposite group.


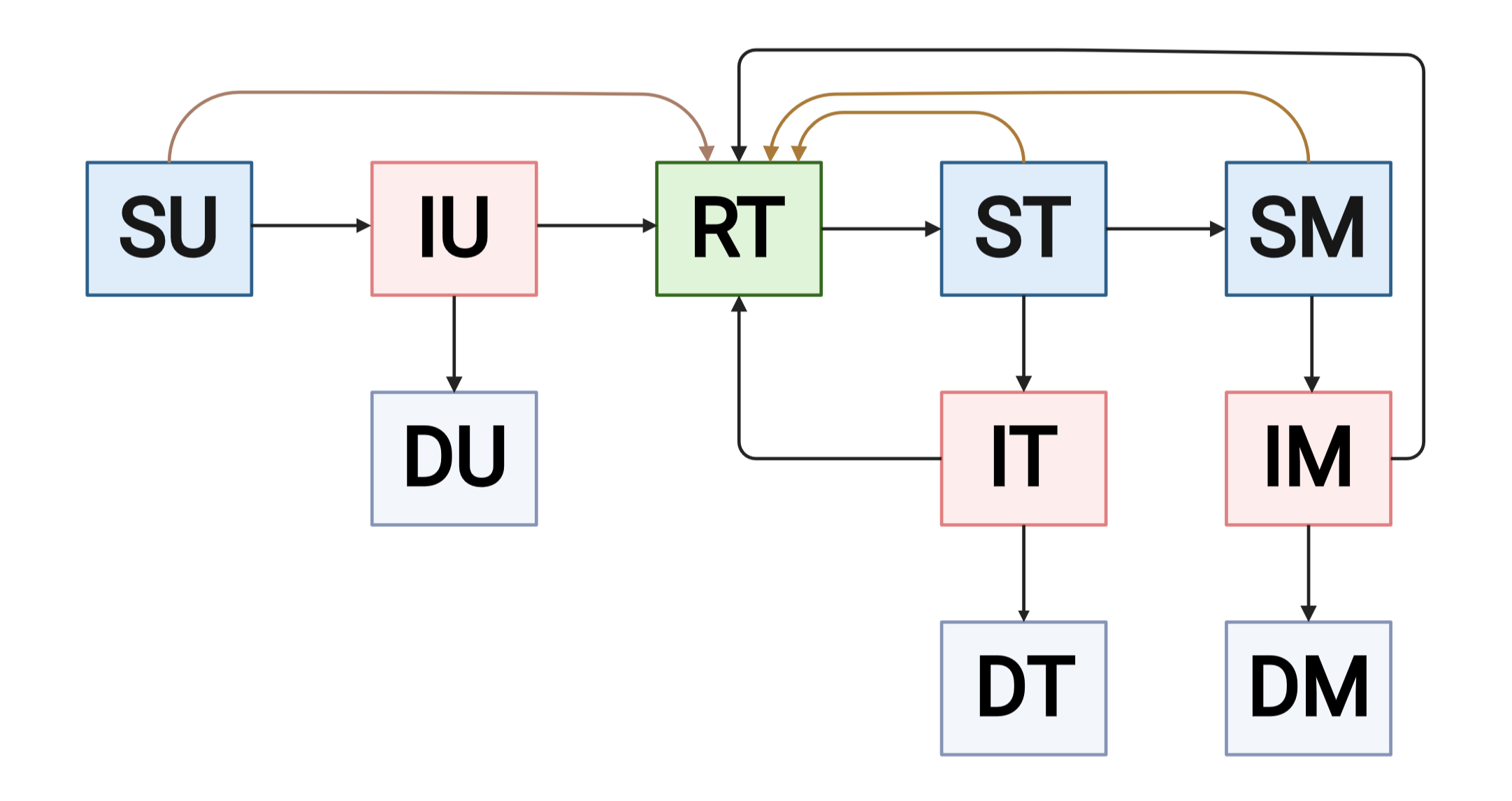


Supplementary Figure 2. Compartmental diagram for vaccination model that tracks status with respect to infection and immune status. The first letter of each compartment name gives the state with respect to the disease transmission process (S=Susceptible, I = Infectious, R = Recovered, D = Deceased) and the second letter of each compartment name gives immune status (U = Unprotective, T = Transmission and Mortality-Reducing, M = Mortality-Reducing alone). Squares are colored based on state with respect to disease. Potential transitions are indicated with arrows. Brown arrows indicate awareness-based vaccination while black arrows indicate disease state transitions. This diagram corresponds to the model described in Equation 3 without groups. Each group contains the compartments and transitions displayed above. Transitions between groups a and b are not possible, but transition rates in one group may be dependent on infection prevalence and mortality in the opposite group.

# Cumulative deaths at different levels of awareness and mixing separation

To better understand the impacts of awareness and mixing separation, we plot cumulative deaths in group *a*, group *b*, and the full population (group *a* and group *b* combined) for the nonpharmaceutical intervention model in Equations 1-2 (Supplementary Figure 3). Epidemic outcomes only vary with separated mixing (Figure 1), which reduces cumulative deaths in group *b* and increases cumulative deaths in group *a* for most of the epidemic. The difference in cumulative deaths between the two groups is maintained across time given uniform awareness, as group *a* experiences a large epidemic with more cumulative infections (Figures 1C, 2E, Supplementary Figure 3C and D). With separated awareness, both groups reach the same cumulative deaths by the end of the epidemic, experiencing epidemics of equivalent sizes with a time delay (Figure 1D) (Supplementary Figure 3D). Mixing and awareness separation have minimal impact on cumulative deaths in the full population; instead, disease burden shifts between group *a* and *b* in an approximately symmetric manner (Supplementary Figure 5A and C).


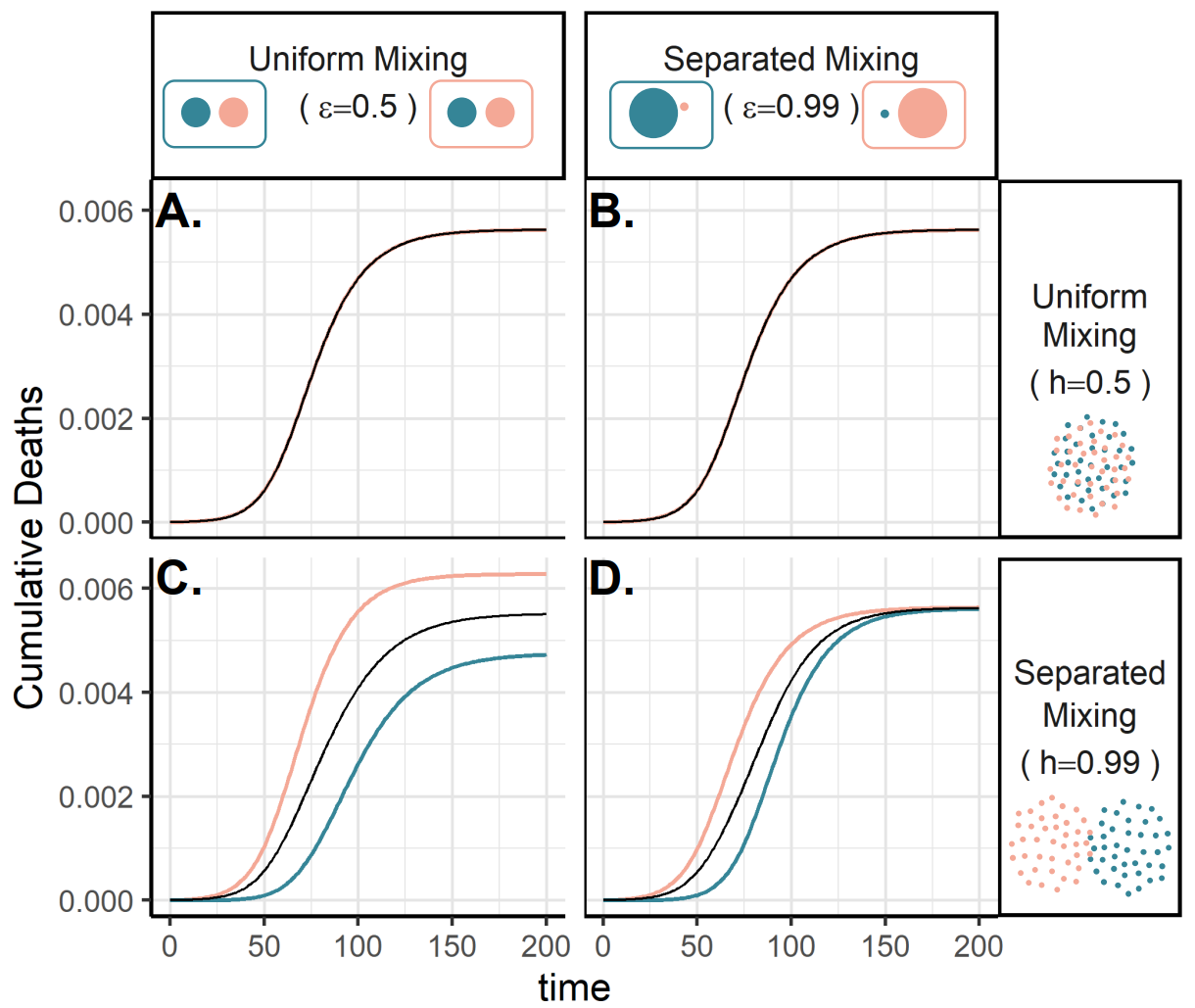


Supplementary Figure 3. Separated mixing causes differences in cumulative deaths between the two groups, which close over time under separated awareness. Plots show cumulative deaths over time in group a (pink), group b (green), and the full population (black) under four scenarios: awareness is uniform (A, C; $\epsilon=0.5$) or separated (B, D; $\epsilon=0.99$); mixing is uniform (A, B; $h=0.5$) or separated (C, D; $h=0.99$). All parameter values are the same as those used in Figure 1. Lines overlap under uniform mixing (top row).

# Intermediate levels of awareness and mixing separation

We further characterize the effects of awareness and mixing separation by comparing intermediate levels of either in the nonpharmaceutical intervention model in Equations 1-2. In Supplementary Figure 4, we plot infections over time for additional combinations of awareness and mixing separation ($\epsilon=0.75, 0.9; h=0.9, 0.97)$, expanding on Figure 1. Supplementary Figure 5 shows how epidemic metrics (total infections, peak infections, and date of infection peak) respond to intermediate levels of awareness and mixing separation by varying one parameter and fixing the other.

Between-group differences given uniform awareness only become apparent at high levels of mixing separation ($h>0.9$) (Supplementary Figures 4, and Supplementary Figure 5B and D). Given separated mixing, total infections and peak infections vary approximately linearly with awareness separation, increasing in group *b* and decreasing in group *a* with greater levels of awareness separation (Supplementary Figure 4, Supplementary Figure 5A and C). Peak and total infections in the full population are approximately constant across different levels of awareness separation, suggesting that disease burden shifts between groups (Supplementary Figure 5A and C). Given uniform awareness, separated mixing reduces peak infections in the full population (Supplementary Figure 5D) because the peaks in the two groups do not coincide (the peak in group *b* occurs approximately 30 days after the peak in group *a*) (Supplementary Figure 5F).

*
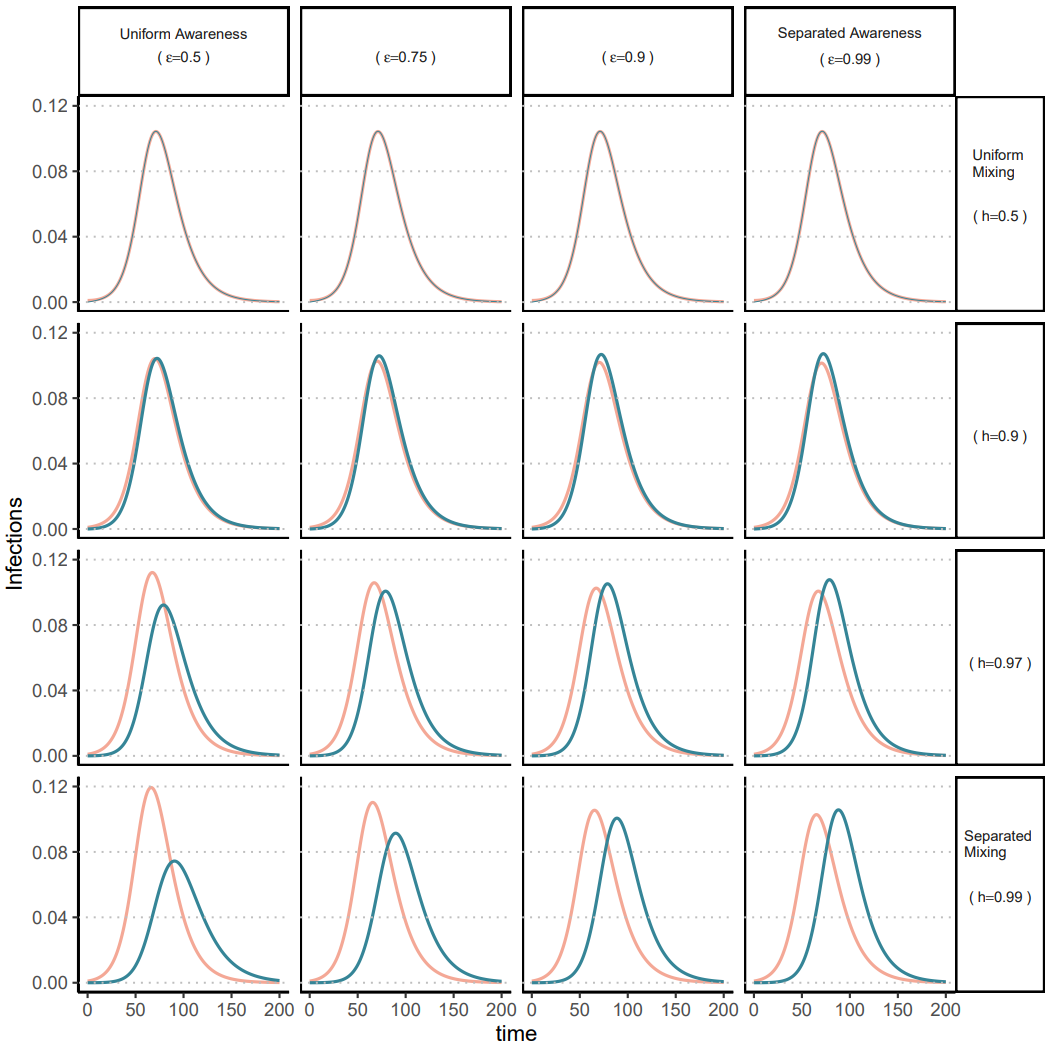
*

Supplementary Figure 4. Infection time series at intermediate levels of mixing and awareness separation demonstrate that a high level of mixing separation is necessary to produce between-group differences in epidemic trajectories. Plots show infections over time in group a (pink) and group b (green) under sixteen scenarios. Awareness varies across the rows between uniform and separated ($\epsilon=0.5$, 0.75, 0.9, 0.99). Mixing varies across the between uniform and separated ($h=0.5$, 0.9, 0.97, 0.99). All other parameter values are the same as those used in Figure 1.


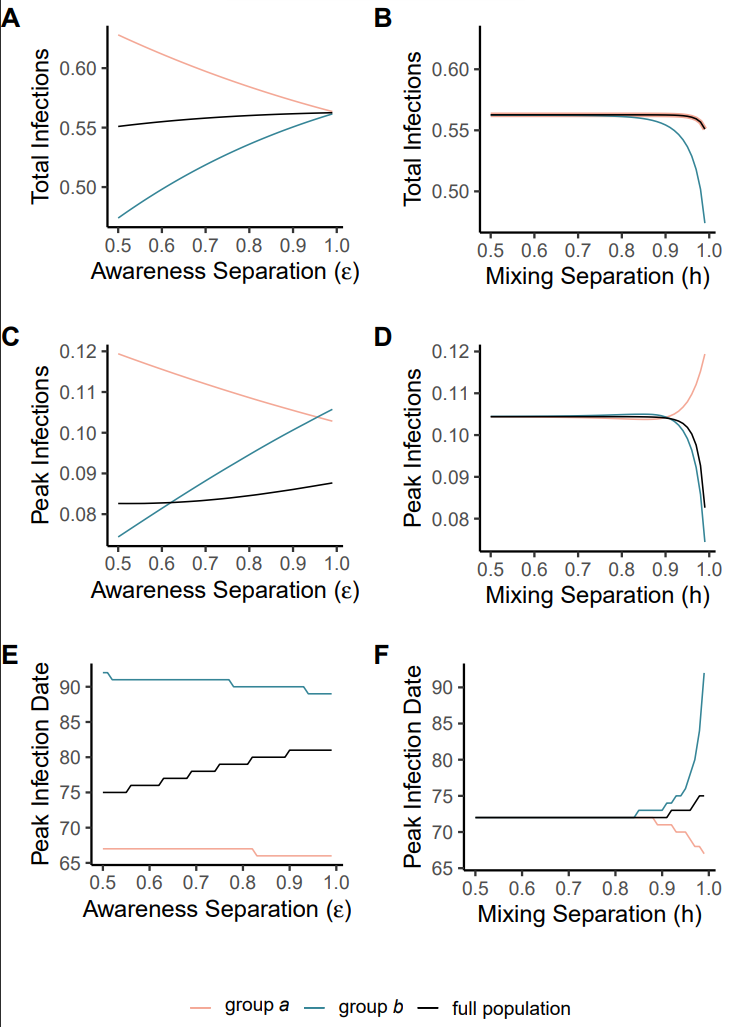


Supplementary Figure 5. Given highly separated mixing, between-group differences in total and peak infection prevalence decrease with increasing awareness separation; at intermediate levels of mixing separation, the groups experience identical epidemic trajectories. Plots show epidemic metrics for group a (pink) group b (green) and the full population (black) across different levels of awareness separation ($\epsilon)$ with separated mixing ($h= 0.99$) (A, C, E) or different levels of mixing separation ($h)$ with uniform awareness ($\epsilon= 0.5$) (B, D, F). The epidemic metrics are total infections across the full epidemic (A, B), peak infection prevalence in a single day (C, D), and the day on which peak infection prevalence occurs (E, F). All other parameter values are the same as those used in Figure 1.

# Separated awareness helps to overcome different types of between-group differences

We demonstrate that the finding in Figure 1 applies across alternative scenarios where the pathogen is introduced in both groups at the same prevalence, but the groups differ in their transmission coefficients ($\beta$), fatality probabilities ($\mu$), or infectious period ($\frac{1}{\rho}$) (Supplementary Figures 4, 5, 6). Note that, when transmission coefficient ($\beta$) varies between groups, contacts between group *a* and group *b* will have transmission coefficient $\sqrt{\beta_{a}\beta_{b}}$, the geometric mean of the transmission coefficient of both groups.

Differences between groups that directly influence force of infection (e.g., variation in transmission coefficient and infectious period) lead to differences in epidemic shape between the groups when mixing is separated (Supplementary Figures 6, 7). Given uniform awareness, epidemic shape is unaffected by mixing separation when group differences do not directly affect the transmission process (e.g., heterogeneity in fatality probabilities) (Supplementary Figure 8). In all scenarios, separated awareness decreases differences in deaths between the two groups, although it may not eliminate differences in epidemic burden. In scenarios where groups have different forces of infections, separated awareness also reduces differences in infections (Supplementary Figures 6, 7). However, separated awareness increases the difference in infections when groups have different fatality probabilities, as observed in the vaccination scenario in the main text (Figures 4, 5).


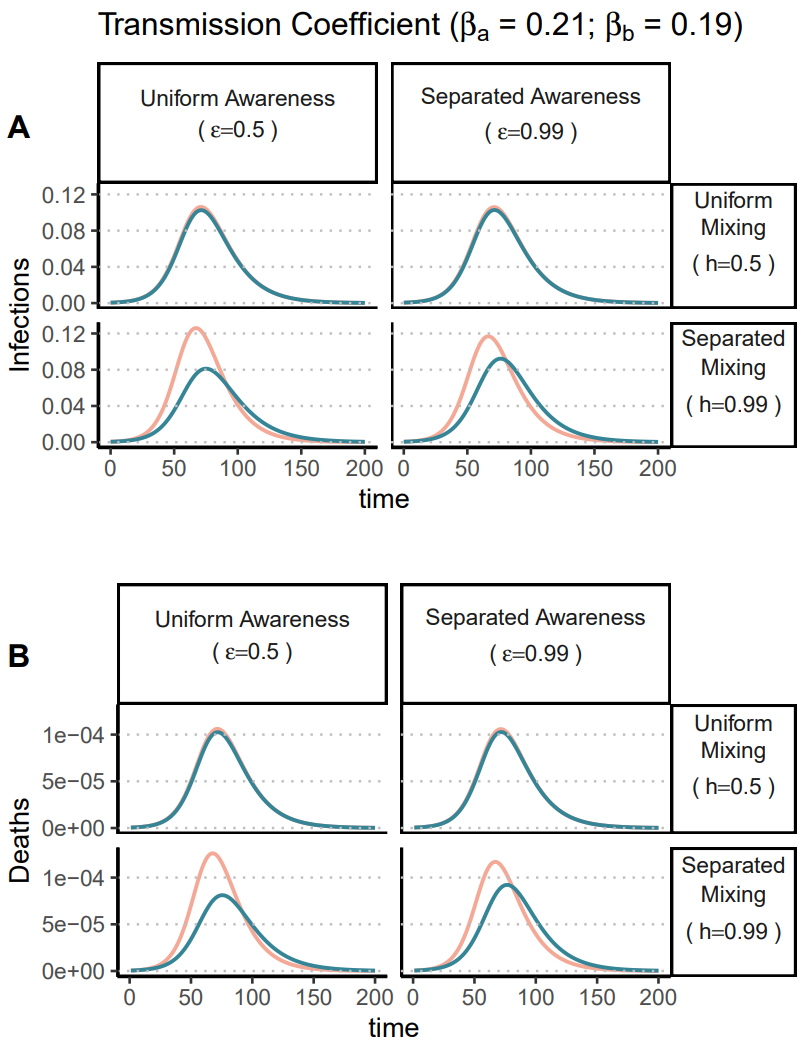


Supplementary Figure 6. Separated awareness reduces differences in epidemic size between groups in epidemic size that arise from differences in transmission rates coupled with separated mixing. Plots of (A) infections and (B) deaths over time in group a (pink) and group b (green). We consider different levels of awareness separation [left column: uniform awareness ($\epsilon=0.5$); right column: separated awareness ($\epsilon=0.99$)] and mixing separation [top row: uniform mixing ($h=0.5$); bottom row: separated mixing ($h=0.99$)]. The groups are initialized so that group a has a greater transmission coefficient than group b ($\beta_{a}=0.21$ and $\beta_{b}=0.19$). We assume the pathogen is introduced in both groups at prevalence $0.0005$. All other parameter values are the same as those used in Figure 1.


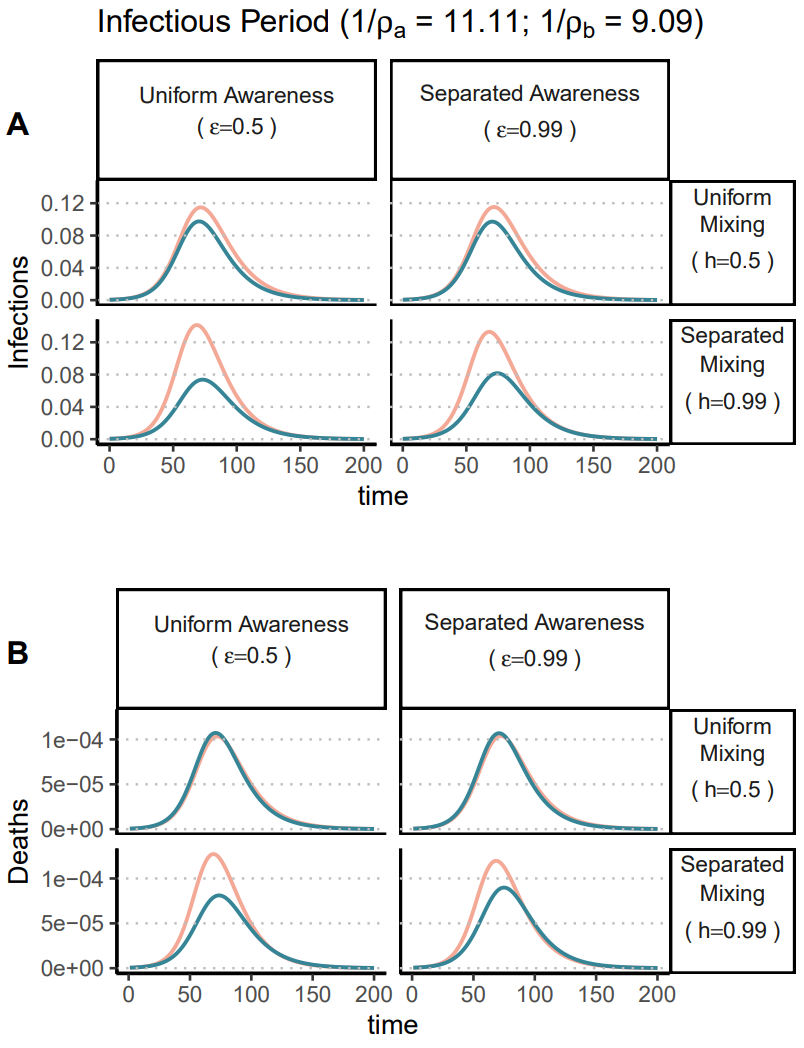


Supplementary Figure 7. Separated awareness reduces differences in epidemic size between groups in epidemic size that arise from differences in infectious period coupled with separated mixing. Plots of (A) infections and (B) deaths over time in group a (pink) and group b (green). We consider different levels of awareness separation [left column: uniform awareness ($\epsilon=0.5$); right column: separated awareness ($\epsilon=0.99$)] and mixing separation [top row: uniform mixing ($h=0.5$); bottom row: separated mixing ($h=0.99$)]. The groups are initialized so that group a has a longer infectious period than group b ($\frac{1}{\rho_{a}}=11.11$ and $\frac{1}{\rho_{b}}=9.09$). We assume the pathogen is introduced in both groups at prevalence $0.0005$. All other parameter values are the same as those used in Figure 1.


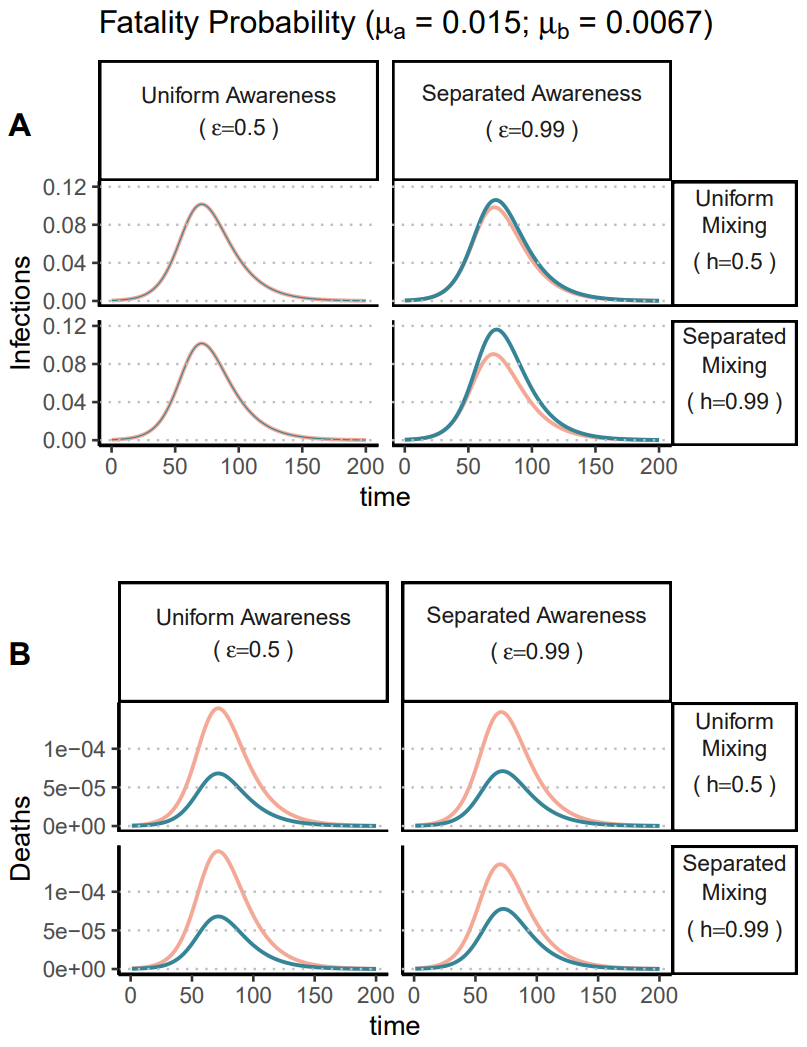


Supplementary Figure 8. Separated awareness reduces differences in mortality between groups arising from differences in their fatality probabilities and causes differences in infections between the groups. Plots of (A) infections and (B) deaths over time in group a (pink) and group b (green). We consider different levels of awareness separation [left column: uniform awareness ($\epsilon=0.5$); right column: separated awareness ($\epsilon=0.99$)] and mixing separation [top row: uniform mixing ($h=0.5$); bottom row: separated mixing ($h=0.99$)]. The groups are initialized so that group a has a higher fatality probability than group b ($\mu_{a}=0.015$ and $\mu_{b}=0.0067$). We assume the pathogen is introduced in both groups at prevalence $0.0005$. All other parameter values are the same as those used in Figure 1.

# Sensitivity to transmissibility, protective measure efficacy, and responsiveness

Supplementary Figure 9 shows that the difference in epidemic size between groups under uniform awareness and separated mixing is sensitive to pathogen transmissibility ($\beta$), the efficacy of protective measures ($\kappa$), and behavioral responsiveness to mortality ($\theta).$In general, the ratio of cases in group a versus group b increases under conditions with reduced transmission due to pathogen characteristics (decreasing $\beta$), or better protective measures (decreasing $\kappa)$ with greater uptake (greater $\theta)$. In these scenarios, both groups experience fewer infections but group b’s response to early awareness of deaths in group a can more dramatically dampen the epidemic. Across the parameter values considered here, separated awareness causes the two groups to have the same epidemic size.


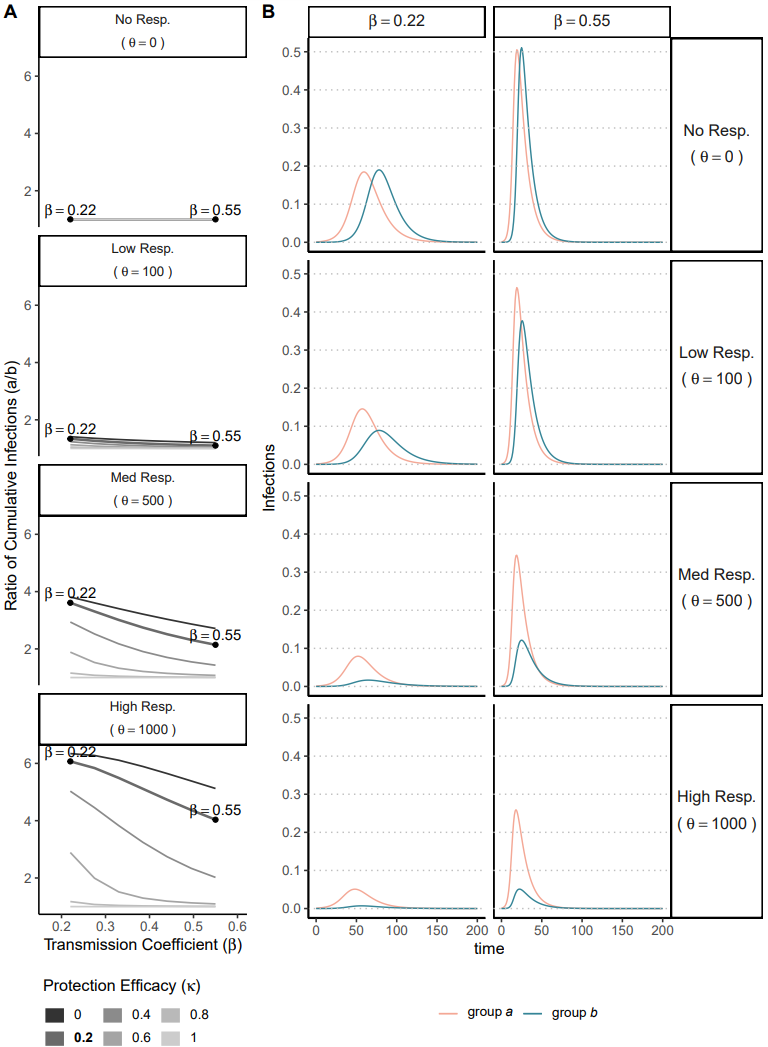


Supplementary Figure 9. The ratio of total infections in group a versus group b increases with reduced pathogen transmissibility, greater protective behavior efficacy, or greater responsiveness. We conduct a sensitivity analysis to determine the effects of transmission coefficient ($\beta)$, protective behavior efficacy ($\kappa$), and responsiveness $(\theta)$ on: (A) the ratio of cumulative infections in group a versus group b and (B) infections over time in groups a and b. All plots assume uniform awareness ($\epsilon=0.5$) and separated mixing ($h=0.99$). The first column of plots, Panel A, shows the ratio of cumulative infections in group a versus group b across different transmission coefficients ($\beta)$, with increasing values on the y-axis indicating a greater cumulative infection burden in group a versus group b. Each line in a given panel indicates a different level of protective measure efficacy ($\kappa$), with darker line color indicating increased protective measure efficacy (i.e., decreased $\kappa$). Plots are arranged from top to bottom by increasing responsiveness (greater values of $\theta$), as indicated by labels at the top of the plots. The second two columns of plots, Panel B, shows infections over time in group a (pink) and group b (green) given different transmission coefficients ($\beta=0.22$or $0.55$) [columns] and responsiveness ($\theta=0, 100, 500$or $1000$) [rows] indicated by facet labels. We annotate Panel A to indicate the parameter values used in Panel B and facilitate comparison between Panel A and Panel B. In particular, the black points correspond to $\kappa=0.2$and $\beta=0.22$or $0.55$ (values given by numerical labels). All other parameter values are the same as those used in Figure 1.

# Long-term memory and fatigue

Supplementary Figure 10 shows how outcomes in both groups vary at intermediate levels of awareness separation. Supplementary Figure 11 shows infection prevalence and cumulative deaths over time in the full population depending on awareness separation, demonstrating that awareness separation may alter the shape of the epidemic without significantly affecting cumulative deaths (as in Supplementary Figure 3).


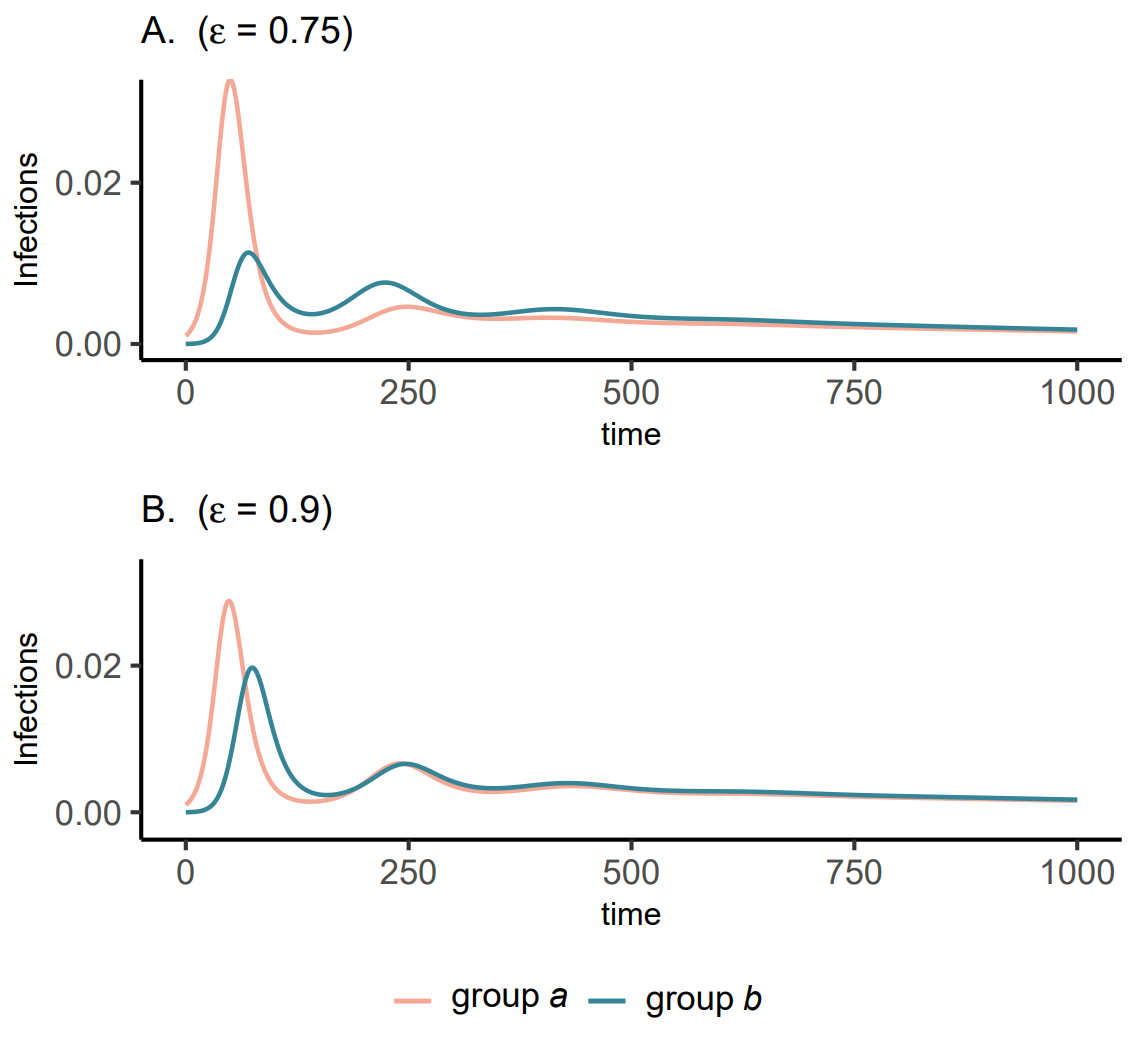


Supplementary Figure 10. At intermediate levels of awareness separation, the first and second waves in group b have similar infection peak sizes. We consider infections in group a (pink) and group b (green) over a longer time period (1000 days) given fatigue and memory. The panels correspond two levels of intermediate awareness: (A) $\epsilon=0.75$ and (B) $\epsilon=0.9$. All other parameter values are the same as those used in Figure 3.


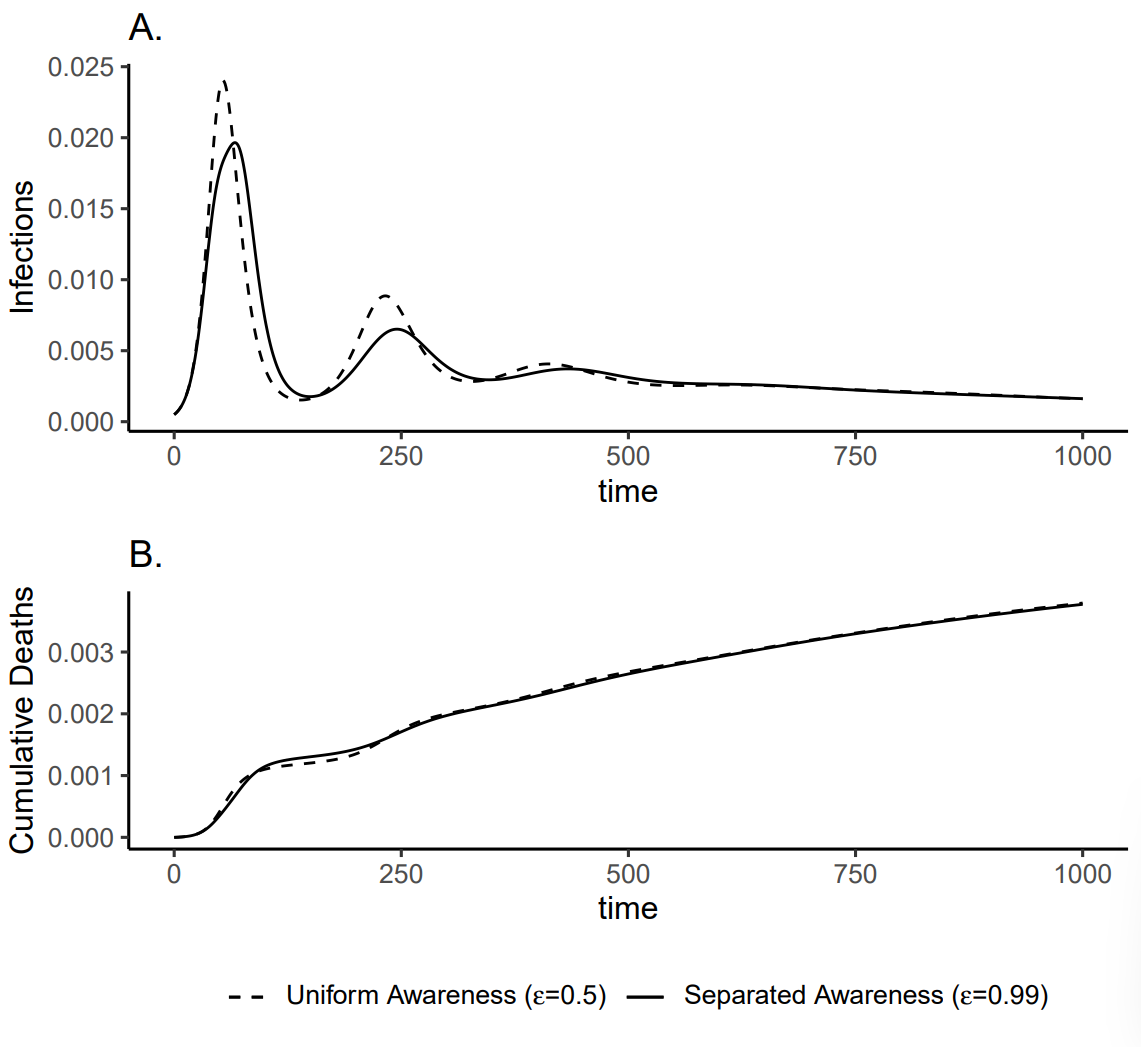


Supplementary Figure 11. In the full population, awareness separation alters the shapes of the first and second waves of infections but does not significantly change cumulative deaths over time. Plots of (A) infections and (B) cumulative deaths over time in the full population given fatigue. We assume mixing separation and consider uniform awareness (dashed lines; $\epsilon=0.5$); and separated awareness (solid lines; $\epsilon=0.99$). All other parameter values are the same as those used in Figure 3.

# Time series with pre-vaccine period

Figure 4 crops time to the post-vaccine period only since the initial wave in an immunologically naïve population is large enough to make visual comparison of the two scenarios (vaccination under separated versus uniform awareness) difficult. Supplementary Figure 12 shows the entire time series, including the pre-vaccine period (t = 0 to t = 200). In the pre-vaccine period, regardless of awareness separation, infection dynamics are identical between groups but deaths are higher in group *a* than group *b* due to a doubly high fatality probability.


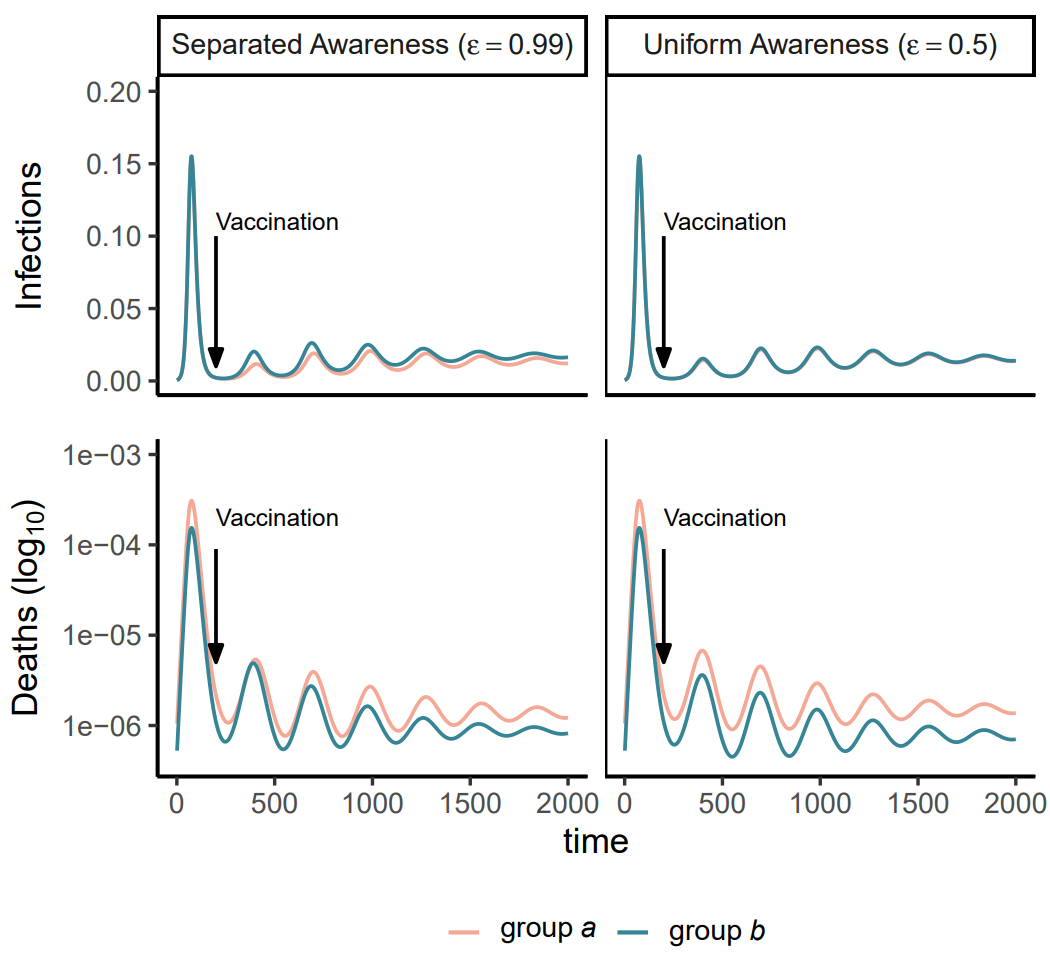


Supplementary Figure 12. Epidemic intensity is greatest in the pre-vaccine period compared to the post-vaccine period. We plot infections (A, B) and log-scaled deaths (C, D) in group a (pink) and group b (green). Vaccination begins at day 200, indicated with vertical arrows. In the post-vaccine period, we compare uniform awareness ($\epsilon=0.5$) (A, C) and separated awareness ($\epsilon=0.99$) (B, D). All parameter values are the same as in Figure 4, but now we include the pre-vaccine period and consider time since pathogen introduction instead of time since the start of vaccination.

# Sensitivity of vaccination outcomes to timing of vaccine introduction

Cumulative deaths in both groups are sensitive to the timing of vaccine introduction ($t_{v})$. Supplementary Figure 13 shows cumulative deaths over time in each group depending on when the vaccine is introduced ($t_{v}$ = 0, 50, 100, or 200). Supplementary Figure 14 shows cumulative deaths, infections, and vaccinations in both groups across different levels of immune protection and awareness separation (following Figure 5), depending on when the vaccine is introduced.

Since cumulative deaths increase the most quickly at the beginning of the epidemic due to the lack of mortality-reducing immunity in the population, early vaccination can decrease cumulative deaths in both groups and narrow the difference in cumulative deaths between the two groups (Supplementary Figure 13, Supplementary Figure 14A-D). The potential for separated awareness to reduce the difference in deaths between both groups is also greatest when vaccination begins earlier because groups can respond to their differences in fatality probabilities more quickly (Supplementary Figure 13, Supplementary Figure 14A-D). Early vaccine introduction may also decrease cumulative infections if immune protection is low to intermediate (Supplementary Figure 14I-L).


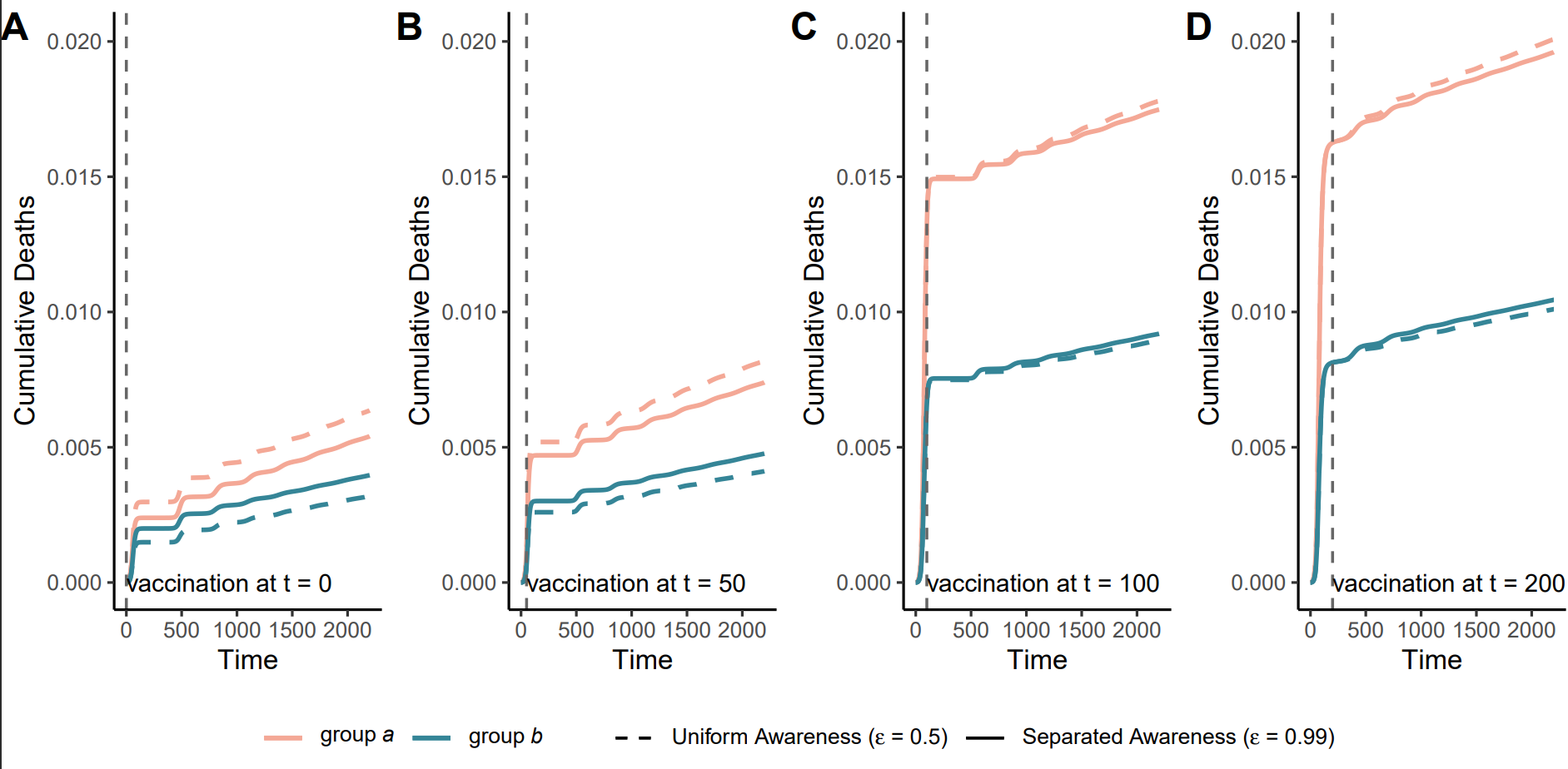


Supplementary Figure 13. Between-group differences in cumulative deaths increase most quickly at the beginning of the epidemic and may be reduced with earlier vaccine introduction. Each plot shows cumulative deaths over time in group a (pink) and group b (green) given consider uniform awareness (dashed lines; $\epsilon=0.5$); and separated awareness (solid lines; $\epsilon=0.99$). The panels are ordered from left to right by later vaccine introduction time (vaccination at $t_{v}$ = 0, 50, 100, or 200) and the dashed vertical line indicates time of vaccine introduction. All other parameter values are the same as in Figure 4, including separated mixing ($h=0.99$).


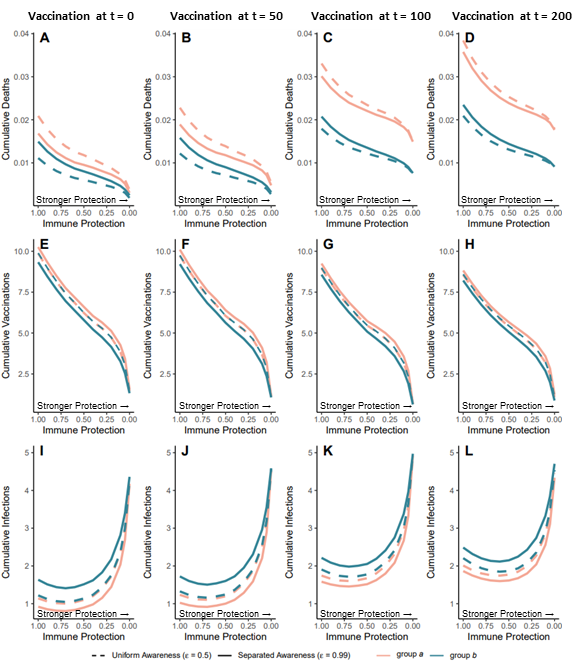


Supplementary Figure 14. Earlier vaccine introduction decreases cumulative deaths in both groups, has minimal impact on cumulative vaccinations, and may decrease cumulative infections if immune protection is low to intermediate. We vary immune protection, defined as transmission-reducing immunity and mortality-reducing immunity, assigning where both parameters the are assigned the same values (κ=ζ). We assume immune protection is equivalent for vaccine- and infection-derived immunity. The x-axis is reversed because smaller values indicate stronger protection. We examine the impacts of stronger immune protection (lower values of $\kappa$ and $\zeta$) on cumulative deaths (A-D), vaccinations (E-H), and infections (I-L) across the entire epidemic (from t=0 to t=2200), depending on awareness separation. The timing of vaccination introduction varies across columns and is set to: $t_{v}$ = 0 (A, E, I); $t_{v}$ = 50 (B, F, J); $t_{v}$ = 100 (C, G, K); and $t_{v}$ = 200 (D, H, L). We compute each quantity for group a (pink) and group b (green) given uniform (dashed lines; $\epsilon=0.5$) or separated (solid lines; $\epsilon=0.99$) awareness. Other parameter values are the same as Figure 4.
